# The Etiology of Pneumonia in HIV-uninfected South African Children: Findings From the Pneumonia Etiology Research for Child Health (PERCH) Study

**DOI:** 10.1097/INF.0000000000002650

**Published:** 2021-08-25

**Authors:** David P. Moore, Vicky L. Baillie, Azwifarwi Mudau, Jeannette Wadula, Tanja Adams, Shafeeka Mangera, Charl Verwey, Christine Prosperi, Melissa M. Higdon, Meredith Haddix, Laura L. Hammitt, Daniel R. Feikin, Katherine L. O’Brien, Maria Deloria Knoll, David R. Murdoch, Eric A.F. Simões, Shabir A. Madhi

**Affiliations:** From the * South African Medical Research Council Vaccines and Infectious Diseases Analytics Research Unit, Faculty of Health Sciences, University of the Witwatersrand, Johannesburg, South Africa; † Department of Paediatrics & Child Health, Chris Hani Baragwanath Academic Hospital and University of the Witwatersrand, South Africa; ‡ Department of Clinical Microbiology and Infectious Diseases, Chris Hani Baragwanath Academic Hospital, National Health Laboratory Service and University of the Witwatersrand, Johannesburg, South Africa; § Department of International Health, International Vaccine Access Center, Johns Hopkins Bloomberg School of Public Health, Baltimore, MD; ¶ Department of Pathology, University of Otago, Christchurch, New Zealand; ‖ Microbiology Unit, Canterbury Health Laboratories, Christchurch, New Zealand; ** Department of Pediatrics, University of Colorado School of Medicine and Center for Global Health, Colorado School of Public Health, Aurora, CO.

**Keywords:** HIV-uninfected, HIV-exposed, pediatric, pneumonia, etiology, PERCH

## Abstract

Pneumonia is the major contributor to under 5 childhood mortality globally. We evaluated the etiology of pneumonia amongst HIV-uninfected South African children enrolled into the Pneumonia Etiology Research for Child Health case-control study.

**Methods:**

Cases, 1–59 months of age hospitalized with World Health Organization clinically defined severe/very severe pneumonia, were frequency-matched by age and season to community controls. Nasopharyngeal-oropharyngeal swabs were analyzed using polymerase chain reaction for 33 respiratory pathogens, and whole blood was tested for pneumococcal autolysin. Cases were also tested for *Mycobacterium tuberculosis*. Population etiologic fractions (EF) of pneumonia with radiologic evidence of consolidation/infiltrate were derived for each pathogen through Bayesian analysis.

**Results:**

Of the 805 HIV-uninfected cases enrolled based on clinical criteria, radiologically confirmed pneumonia was evident in 165 HIV-exposed, -uninfected, and 246 HIV-unexposed children. In HIV-exposed and HIV-unexposed children, respiratory syncytial virus was the most important pathogen with EFs of 31.6% [95% credible interval (CrI), 24.8%–38.8%] and 36.4% (95% CrI, 30.5%–43.1%), respectively. *M. tuberculosis* contributed EFs of 11.6% (95% CrI, 6.1%–18.8%) in HIV-exposed and 8.3% (95% CrI, 4.5%–13.8%) in HIV-unexposed children, including an EF of 16.3% (95% CrI, 6.1%–33.3%) in HIV-exposed children ≥12 months of age. Bacteremia (3.0% vs. 1.6%) and case fatality risk (3.6% vs. 3.7%) were similar in HIV-exposed and HIV-unexposed children.

**Conclusions:**

Vaccination strategies targeting respiratory syncytial virus should be prioritized for prevention of pneumonia in children. Furthermore, interventions are required to address the high burden of tuberculosis in the pathogenesis of acute community-acquired pneumonia in settings such as ours.

## Abstract 2

Supplemental Digital Content is available in the text.

Despite an overall reduction in under 5 childhood deaths since 1990, pneumonia remains the leading cause of mortality in children 1–59 months of age globally,^1^ including in South Africa.^2^ The burden of pneumonia in South Africa is further exacerbated by the high prevalence of exposure to human immunodeficiency virus type 1 (HIV) in utero, which affects 30% of the approximately 1.0 million newborns annually.^3,4^ The mother-to-child HIV vertical transmission rate in South Africa has, however, declined from 9.6% in 2008 to <2% in the current era of increased access to antiretroviral treatment.^5^ Nevertheless, infants born to HIV-infected women, even if protected against HIV acquisition from their mothers, have increased susceptibility to infection-related morbidity and mortality, especially during infancy. This includes a 2- to 3-fold heightened susceptibility to invasive pneumococcal disease and respiratory virus-associated pneumonia during the first 6 months of life in HIV-exposed uninfected compared with HIV-unexposed infants.^6^ Furthermore, HIV-exposed infants may be at increased risk for disease due to opportunistic pathogens, such as *Pneumocystis jirovecii* and human cytomegalovirus.^6–9^

Previous studies of pneumonia etiology in HIV-uninfected South African children under 5 years of age, conducted in the era before *Haemophilus influenzae* type b (Hib) and pneumococcal polysaccharide-protein conjugate vaccines were incorporated into the National Expanded Program on Immunization, in 1999 and 2009, respectively, indicated that respiratory viruses, pneumococcus and Hib were the predominant organisms leading to severe pneumonia requiring hospitalization.^10,11^ These studies did not, however, systematically evaluate for differences in etiology of pneumonia between HIV-exposed uninfected and HIV-unexposed children.

In the era of molecular diagnostic techniques for the identification of respiratory viral infections, and postintroduction of Hib and pneumococcal conjugate vaccine (PCV), respiratory viruses have been identified in 78% of South African children hospitalized with lower respiratory tract infection.^12^

This study evaluated the pathogen profiles for pneumonia etiology among hospitalized HIV-uninfected, including HIV-exposed, children in Soweto, South Africa, as part of the multicenter Pneumonia Etiology Research for Child Health (PERCH) study. A companion paper,^13^ details the pneumonia etiology profiles in HIV-infected children enrolled at the South African PERCH site.

## MATERIALS AND METHODS

### Location

Enrollment into the PERCH study in South Africa occurred at Chris Hani Baragwanath Academic Hospital, which is a public sector health care facility, and was the only hospital serving the majority of Soweto’s population at the time of the study. Soweto is located 1600 meters above sea level, has a summer rainfall pattern, and an autumn/winter respiratory illness season which lasts from March through August.^14,15^ Further detail regarding the study catchment area is included in Supplemental Digital Content 1, http://links.lww.com/INF/D829.

Pneumonia contributed 11.7% of under 5 deaths in South African children in 2012/2013 with a national average case fatality rate of 3.8%.^16^ The antenatal HIV seroprevalence rate in Gauteng Province in 2012/2013 was 34.0%, and the mother-to-child vertical transmission rate was 2.2% [95% confidence interval (CI), 1.3%–3.1%].^5^ Health care is provided free of charge to all children under 6 years of age attending public sector health facilities in South Africa.^17^

### Participants

Eligibility and exclusion criteria for PERCH case and control selection have been described.^18^ In this analysis, we included HIV-uninfected children between the ages of 1 and 59 months, hospitalized with signs of WHO-defined severe/very severe pneumonia (cases).^19^ HIV-uninfected controls were frequency-matched to cases according to age-stratification (1–5, 6–11, 12–23 and 24–59 months), and all participants were resident in the study catchment area. Community control selection procedures are described in Supplemental Digital Content 1, http://links.lww.com/INF/D829.

### Clinical Procedures

Enrollment occurred through active surveillance in the hospital pediatric admissions ward for potentially eligible cases. Once consented and enrolled, cases were evaluated at baseline, 24 and 48 hours, and again on the day of hospital discharge, to monitor changes in clinical status. Cases were evaluated again after hospital discharge, at least 30 days subsequent to the date of enrollment. All community controls were assessed once, on the day on which they presented to the research clinic at Chris Hani Baragwanath Academic Hospital.

### Specimen Collection and Laboratory Methods

Standardized specimen collection and laboratory procedures were followed in cases and controls, as previously described,^20–23^ and are detailed in Supplemental Digital Content 1, http://links.lww.com/INF/D829. A blood culture and a chest radiograph was obtained from cases as part of the routine diagnostic work-up.^24^ Study-specific specimens obtained from cases and controls included nasopharyngeal, oropharyngeal (NP/OP) swabs for multiplex real-time polymerase chain reaction (PCR) to detect 33 respiratory pathogens (Fast Track Diagnostics Respiratory Pathogens 33 test (FTD-33), Fast Track Diagnostics, Sliema, Malta), whole blood for detection of pneumococcal autolysin (*lytA*) by PCR,^25^ and serum for antibiotic activity.^26^ Age-appropriate HIV testing was also done, with consent, in all participants. HIV-exposure status was determined as detailed in Supplemental Digital Content 1, http://links.lww.com/INF/D829.

### Statistical Analysis

Descriptive analyses of clinical and laboratory measures, reporting percentages in subgroups (stratified by case/control and HIV-exposure status, as well as by disease severity and radiologic findings amongst cases) were undertaken, and proportions were compared using logistic regression, adjusting for age (in months). Medians and interquartile range were used to describe continuous data. In instances where numerous comparisons were done, *P* values were adjusted using the Benjamini-Hochberg method.^27^ Two-sided *P* values < 0.05 were considered to be statistically significant.

Predefined organism-specific thresholds for defining high load that best distinguished between cases and controls for NP/OP swab FTD-33 PCR, and *lytA* on whole blood PCR from the PERCH foundational analyses have been published,^25,28–30^ and were applied to cytomegalovirus, *H. influenzae*, *P. jirovecii* and *Streptococcus pneumoniae* in the current analysis. Conditional logistic regression of respiratory pathogen prevalence in the upper respiratory tracts (for all tested potential pathogens) and whole blood (for pneumococcus only) of cases compared with controls, adjusting for age (in months) and all other pathogens, was used to derive the adjusted odds of each organism being associated with case status. This was integrated into analyses of etiology in addition to other specimens and laboratory tests from cases and controls, which was undertaken using a Bayesian approach through the PERCH Integrated Analysis (PIA) that also accounted for sensitivity and specificity of all measurements.^31,32^ The PIA permitted assignation of pathogen etiology fractions (EFs), including the proportion of cases with no identifiable pathogen. Further detail regarding the PIA model is included in Supplemental Digital Content 1, http://links.lww.com/INF/D829.

Analyses were performed using R version 3.3.3,^33^ SAS 9.4 (SAS Institute, Cary NC), and JAGS 4.2.0 (http://mcmc-jags.sourceforge.net/). For the PIA output, an open-source R software package, the Bayesian Analysis Kit for Etiology Research, was developed specifically for PERCH. The Bayesian Analysis Kit for Etiology Research package is available at http://zhenkewu.com/baker/.

The overarching PERCH paper reported on the etiology of pneumonia in HIV-uninfected children with radiologically confirmed pneumonia at each of the study sites.^34^ The current paper focuses on the South African cohort and presents the pneumonia etiology estimates for HIV-uninfected children, stratified by HIV-exposure status, age and pneumonia severity. The methodology in the current analysis differs from the overarching PERCH paper through its use of a higher sensitivity prior estimate (20%–50%) for *Mycobacterium tuberculosis* (*Mtb*) than was used for the all-site combined analysis (in which the sensitivity prior for *Mtb* was 10%–30%). The higher sensitivity prior estimate adopted in the South African site-specific analysis was chosen because more intensive screening for *Mtb* was conducted at the site,^23^ and because the site has a high burden of tuberculosis as outlined in Supplemental Digital Content 1, http://links.lww.com/INF/D829.

### Ethical Considerations

The Human Research Ethics Committee of the University of the Witwatersrand (M101129) and the Institutional Review Board of the Johns Hopkins Bloomberg School of Public Health approved the study. Parents or legal guardians of all cases and controls provided written consent for participation in PERCH.

## RESULTS

### Study Participants

Between August 17, 2011, and September 4, 2013, 920 cases and 964 controls were enrolled in South Africa, including 805 (87.5%) and 828 (85.9%), respectively, who were HIV-uninfected. Two hundred ninety-eight (37.0%) of these 805 cases were HIV-exposed, 465 (57.8%) were HIV-unexposed, and 42 (5.2%) had undetermined HIV-exposure status. Amongst the 828 controls, 225 (27.2%) were HIV-exposed, 583 (70.4%) were HIV-unexposed, and 20 (2.4%) had undetermined HIV-exposure status (Supplemental Digital Content 2; http://links.lww.com/INF/D830). Cases had 1.64-fold (95% CI, 1.32–2.03) higher odds of being HIV-exposed compared with controls; *P* < 0.001 (Table 1).

**TABLE 1.** Demographic and Clinical Characteristics of HIV-uninfected Cases and Controls Enrolled Into PERCH at the South African Site

Characteristic	All Cases (n = 805)	CXR + Cases (n = 435/771)	All Controls (n = 828)	OR (95% CI); Adjusted *P* Value*	
				All Cases Compared With Controls	CXR+ Cases Compared With Controls
Age (months)					
Median age (IQR)	5.0 (2.0–12.0)	5.0 (2.0–11.0)	8.0 (4.0–16.0)	0.97 (0.96–0.98); <0.001	0.96 (0.95–0.98); <0.001
Sex					
Female	377/805 (46.8)	222/435 (51.0)	424/828 (51.2)	0.84 (0.69–1.02); 0.124	0.99 (0.78–1.26); 0.973
Respiratory tract illness (controls only)†	-	-	44/828 (5.3)	-	-
HIV-exposure status‡					
HIV-unexposed	465/805 (57.8)	246/435 (56.6)	583/828 (70.4)	Ref	Ref
HIV-exposed	298/805 (37.0)	165/435 (37.9)	225/828 (27.2)	1.64 (1.32–2.03); <0.001	1.72 (1.33–2.22); <0.001
Unknown	42/805 (5.2)	24/435 (5.5)	20/828 (2.4)	2.73 (1.56–4.75); <0.001	3.02 (1.61–5.66); 0.002
Anthropometry					
WAZ ≥−2	586/800 (73.2)	295/430 (68.6)	784/821 (95.5)	Ref	Ref
WAZ ≥−3 to <−2	105/800 (13.1)	64/430 (14.9)	27/821 (3.3)	5.25 (3.37–8.15); <0.001	6.28 (3.90–10.12); <0.001
WAZ <−3	109/800 (13.6)	71/430 (16.5)	10/821 (1.2)	13.57 (7.02–26.23); <0.001	17.20 (8.71–34.00); <0.001
Socioeconomic status					
Lowest tier	104/805 (12.9)	63/435 (14.5)	31/823 (3.8)	3.55 (2.22–5.66); <0.001	4.45 (2.63–7.51); <0.001
Low-to-mid tier	224/805 (27.8)	130/435 (29.9)	175/823 (21.3)	1.43 (1.05–1.94); 0.043	1.71 (1.18–2.49); 0.011
Mid-to-upper tier	340/805 (42.2)	174/435 (40.0)	461/823 (56.0)	0.86 (0.66–1.13); 0.376	0.90 (0.64–1.27); 0.643
Upper tier	137/805 (17.0)	68/435 (15.6)	156/823 (19.0)	Ref	Ref
Immunization status					
BCG immunization	742/748 (99.2)	401/405 (99.0)	809/812 (99.6)	0.47 (0.12–1.93); 0.376	0.36 (0.08–1.68); 0.262
DTP-Hib immunization up-to-date§	487/752 (64.8)	264/408 (64.7)	586/811 (72.3)	0.76 (0.61–0.94); 0.027	0.77 (0.59–0.99); 0.073
PCV immunization up-to-date¶	505/752 (67.2)	272/408 (66.7)	602/811 (74.2)	0.60 (0.47–0.75); <0.001	0.57 (0.44–0.75); <0.001
Measles immunization up-to-date‖	217/750 (28.9)	114/407 (28.0)	352/812 (43.3)	0.84 (0.62–1.13); 0.345	0.81 (0.56–1.17); 0.332
CRP					
Median, mg/L (IQR)	12.0 (2.8–39.9)	16.9 (4.7–51.0)	1.0 (0.3–3.5)	1.16 (1.11–1.21); <0.001	1.17 (1.12–1.22); <0.001
≥40, mg/L	196/783 (48.8)	132/422 (31.3)	0/151 (0.0)	N/E	N/E
Prior exposure to medications					
Serum antibiotic activity	375/768 (48.8)	224/413 (54.2)	8/764 (1.0)	89.92 (44.07–183.49); <0.001	111.85 (53.59–231.90); <0.001

*Odds ratio adjusted by age (in months) and season, and derived by logistic regression analysis. *P* values adjusted using the Benjamini-Hochberg method.

Respiratory tract illness in PERCH controls was defined as presence of cough or runny nose, or if a child had (1) at least 1 of ear discharge, wheezing or difficulty breathing and (2) either a measured temperature of >38.0°C within the previous 48 hours or a history of sore throat.

‡HIV-exposure status was attributed as described in the Supplementary Appendix.

§Complete vaccination defined as receipt of ≥3 doses.

¶Complete vaccination defined based on number of doses, and age at first dose, or age at PCV introduction in South Africa: ≥3 doses, or 2 doses if there were at least 8 weeks between doses and the child was <9 months of age at enrollment or >12 months of age at the time of first dose, or ≥1 dose if the age at any of the doses, or age at PCV introduction, was ≥24 months.

‖Complete vaccination defined as receipt of at least one dose, restricted to children aged ≥10 months.

BCG indicates Bacillus Calmette-Guérin; CI, confidence interval; CRP, C-reactive protein; CXR+, radiologically confirmed pneumonia; DTP, diphtheria, tetanus, pertussis; N/E, no estimate; Ref, referent; WAZ, weight-for-age Z-score.

Overall, cases were younger than controls, more likely to be under-vaccinated with PCV (67.2% vs. 74.2%; *P* < 0.001) and more likely to belong to the lowest socioeconomic stratum (12.9% vs. 3.8%; *P* < 0.001; Table 1). Cases were also more likely to be severely underweight-for-age (13.6% vs. 1.2%; *P* < 0.001) and had a higher prevalence of serum antibiotic activity compared with controls (48.8% vs. 1.0%; *P* < 0.001; Table 1). Bacillus Calmette-Guérin vaccination coverage was high (≥99.0%) in cases and controls. Similar differences were observed between cases and controls when stratified by HIV-exposure status (Supplemental Digital Content 3, http://links.lww.com/INF/D831 and Supplemental Digital Content 4, http://links.lww.com/INF/D832).

### Case Characteristics

Of 771 cases with interpretable chest radiographs, 435 (56.4%) had radiologically confirmed pneumonia, and overall 259 (32.2%) had very severe pneumonia according to the pre-2013 WHO clinical criteria (Supplemental Digital Content 5, http://links.lww.com/INF/D833). Cases with radiologically confirmed pneumonia compared with those with a normal chest radiograph, were more likely to be severely underweight-for-age (16.5% vs. 8.1%; *P* < 0.001), present with fever (68.5% vs. 50.4%; *P* < 0.001) and have a longer hospital stay; but less likely to have wheeze on chest auscultation (29.0% vs. 42.6%; *P* < 0.001) (Supplemental Digital Content 5, http://links.lww.com/INF/D833).

There were 20 in-hospital deaths among HIV-uninfected children, with the case fatality rate being similar in HIV-exposed (8/298, 2.7%) and HIV-unexposed children (11/464, 2.4%) (Supplemental Digital Content 5, http://links.lww.com/INF/D833). Overall, fatal cases were more likely to present with lethargy (40.0% vs. 4.2%; *P* < 0.001) and difficulty feeding (30.0% vs. 3.1%; *P* < 0.001) compared with survivors.

### Microbiologic Results in HIV-uninfected Cases

Clinically significant pathogens were isolated in 17 (2.1%) of 802 blood cultures submitted in the cases, with Gram-negative organisms (n = 10, 58.8%) predominating over Gram-positive species (n = 7, 41.2%). The single most common blood culture pathogen was, however, *Staphylococcus aureus* (n = 5; 29.4% of all significant isolates) (Supplemental Digital Content 6, http://links.lww.com/INF/D834). Nine (52.9%) of the 17 clinically significant blood culture isolates were in children with radiologically confirmed pneumonia, 5 (29.4%) in children with normal chest radiographs and 3 (17.6%) in children with uninterpretable chest radiographs. Bacteremia in children with radiologically confirmed pneumonia was present in 3.0% (5/164) of HIV-exposed children, and in 1.6% (4/245) of those that were HIV-unexposed, *P* = 0.494. Detailed descriptions of children with Gram-negative bacteremia, those with microbiologically confirmed pneumococcal infection (n = 4) and microbiological results of children that underwent lung aspiration are presented in Supplemental Digital Content 1, http://links.lww.com/INF/D829.

Twenty-three (2.9%) of the cases cultured *Mtb* on respiratory specimens, and 14.9% (51 of 342 cases) had a reactive tuberculin skin test suggestive of underlying *Mtb* infection (Supplemental Digital Content 6, http://links.lww.com/INF/D834).

### Comparison of NP/OP FTD-33 and Whole Blood Pneumococcal PCR Results Between HIV-uninfected Cases and Controls, Stratified by HIV-exposure Status

In both HIV-exposed and HIV-unexposed children, the presence of any respiratory viral organism detected by PCR on NP/OP swab was significantly associated with case-status (Tables 2 and 3). Furthermore, in HIV-exposed children, RSV [adjusted odds ratio (aOR), 19.03], influenza A (aOR, 5.10), and nontype b *H. influenzae* (aOR, 1.72) on NP/OP PCR testing and high load pneumococcus detected in whole blood by PCR (aOR 3.34) were associated with radiologically confirmed pneumonia case-status; Table 2. Organisms associated with case-status in HIV-unexposed children with radiologically confirmed pneumonia compared with controls were generally similar but more organisms were significantly associated because sample size was larger (Table 3). The additional organisms included parainfluenza virus 1 (aOR, 19.15), influenza B (aOR, 9.07), parainfluenza virus 3 (aOR, 6.90), *Bordetella pertussis* (aOR, 6.85), and high-density *P. jirovecii* (aOR, 2.32); Table 3.

**TABLE 2.** Conditional Odds Ratios in the Comparison Between All Cases, Cases With Radiologically Confirmed Pneumonia, and Controls: HIV-exposed Children

Pathogen	All Cases	CXR+ Cases	Controls	Conditional Odds Ratio (95% CI)*	
				All Cases vs. Controls	CXR+ Cases vs. Controls
Any nonviral pathogen	277/297 (93.3)	153/164 (93.3)	210/224 (93.8)	0.93 (0.45–1.92)	0.94 (0.40–2.22)
Any nonviral pathogen, above cutoff density threshold†	236/297 (79.5)	130/164 (79.3)	191/224 (85.3)	0.60 (0.37–0.98)	0.58 (0.33–1.03)
Bacteria					
*Bordetella pertussis*	6/297 (2.0)	3/164 (1.8)	1/224 (0.4)	5.51 (0.62–49.22)	4.55 (0.38–54.98)
*Chlamydophila pneumonia*	6/297 (2.0)	5/164 (3.0)	3/224 (1.3)	2.32 (0.47–11.48)	3.20 (0.56–18.40)
*Haemophilus influenzae* type b	4/297 (1.3)	2/164 (1.2)	2/224 (0.9)	2.45 (0.37–16.07)	3.59 (0.41–31.74)
*Haemophilus influenzae* type b ≥ threshold density‡	2/297 (0.7)	1/164 (0.6)	0/224 (0.0)	0.76 (0.42-1.36)	1.08 (0.55-2.12)
Nontype b *Haemophilus influenza*	148/297 (49.8)	97/164 (59.1)	111/224 (49.6)	1.17 (0.76–1.79)	**1.72 (1.01–2.93**)
Nontype b *Haemophilus influenzae* ≥ threshold density‡	85/297 (28.6)	57/164 (34.8)	57/224 (25.4)	1.12 (0.70–1.79)	1.36 (0.78–2.37)
*Moraxella catarrhalis*	172/297 (57.9)	99/164 (60.4)	149/224 (66.5)	0.80 (0.52–1.24)	0.81 (0.47–1.40)
*Mycoplasma pneumoniae*	2/293 (0.7)	1/161 (0.6)	2/224 (0.9)	1.23 (0.10–15.58)	0.69 (0.01–46.07)
*Streptococcus pneumoniae*	185/297 (62.3)	115/164 (70.1)	155/224 (69.2)	0.82 (0.52–1.28)	1.06 (0.59–1.90)
*Streptococcus pneumoniae* ≥ threshold density§	30/297 (10.1)	19/164 (11.6)	23/224 (10.3)	0.83 (0.41–1.69)	0.97 (0.42–2.25)
Vaccine-type *Streptococcus pneumoniae*¶	13/297 (4.4)	10/164 (6.1)	8/224 (3.6)	0.89 (0.29–2.74)	1.45 (0.42–4.93)
Nonvaccine-type *Streptococcus pneumoniae*¶	18/297 (6.1)	10/164 (6.1)	15/225 (6.7)	0.88 (0.38–2.06)	0.84 (0.29–2.44)
*Streptococcus pneumoniae* in whole blood	19/296 (6.4)	11/163 (6.7)	23/225 (10.2)	0.90 (0.44–1.86)	0.95 (0.39–2.35)
*Streptococcus pneumoniae* in whole blood ≥ threshold density‖	16/296 (5.4)	11/163 (6.7)	11/225 (4.9)	2.20 (0.85–5.62)	**3.34 (1.14–9.75**)
Salmonella spp	0/297 (0.0)	0/164 (0.0)	0/224 (0.0)	N/E	N/E
*Staphylococcus aureus*	80/297 (26.9)	36/164 (22.0)	40/224 (17.9)	1.49 (0.90–2.47)	1.05 (0.54–2.05)
Fungal species					
*Pneumocystis jirovecii*	36/297 (12.1)	25/164 (15.2)	21/224 (9.4)	1.12 (0.58–2.18)	1.53 (0.71–3.30)
*Pneumocystis jirovecii* ≥ threshold density**	22/297 (7.4)	14/164 (8.5)	5/224 (2.2)	**3.07 (1.06–8.93**)	3.14 (0.96–10.24)
Viruses					
Any viral pathogen	244/297 (82.2)	142/164 (86.6)	158/224 (70.5)	**1.93 (1.26–2.96**)	**2.70 (1.55–4.67**)
Any viral pathogen, above cutoff density threshold†	234/297 (78.8)	138/164 (84.1)	141/224 (62.9)	**2.25 (1.50–3.37**)	**3.22 (1.92–5.41**)
Adenovirus	35/293 (11.9)	25/161 (15.5)	38/224 (17.0)	0.76 (0.42–1.36)	1.08 (0.55–2.12)
Human cytomegalovirus	99/293 (33.8)	61/161 (37.9)	74/224 (33.0)	1.24 (0.80–1.90)	1.51 (0.89, 2.55)
Human cytomegalovirus ≥ threshold density††	56/293 (19.1)	39/161 (24.2)	32/224 (14.3)	1.15 (0.66–2.03)	1.55 (0.80–2.99)
Coronavirus 229	0/293 (0.0)	0/161 (0.0)	0/224 (0.0)	N/E	N/E
Coronavirus 43	7/293 (2.4)	3/161 (1.9)	17/224 (7.6)	0.35 (0.13–0.94)	0.28 (0.07–1.11)
Coronavirus 63	9/293 (3.1)	6/161 (3.7)	3/224 (1.3)	2.67 (0.64–11.14)	4.34 (0.93–20.23)
Coronavirus HKU	8/293 (2.7)	6/161 (3.7)	3/224 (1.3)	2.34 (0.53–10.35)	3.42 (0.60–19.42)
Influenza A	12/293 (4.1)	8/161 (5.0)	3/224 (1.3)	**4.61 (1.17–18.10**)	**5.10 (1.13–23.10**)
Influenza B	1/293 (0.3)	1/161 (0.6)	1/224 (0.4)	1.96 (0.10–39.46)	5.19 (0.24–110.22)
Influenza C	3/297 (1.0)	2/164 (1.2)	0/224 (0.0)	N/E	N/E
Human bocavirus	31/293 (10.6)	18/161 (11.2)	21/224 (9.4)	1.46 (0.73–2.94)	1.28 (0.54–3.03)
Human metapneumovirus A/B	23/293 (7.8)	14/161 (8.7)	7/224 (3.1)	0.76 (0.42–1.36)	1.08 (0.55–2.12)
Parainfluenza virus 1	6/293 (2.0)	1/161 (0.6)	0/224 (0.0)	N/E	N/E
Parainfluenza virus 2	0/293 (0.0)	0/161 (0.0)	2/224 (0.9)	N/E	N/E
Parainfluenza virus 3	9/293 (3.1)	6/161 (3.7)	5/224 (2.2)	2.06 (0.63–6.68)	3.02 (0.82–11.11)
Parainfluenza virus 4	7/293 (2.4)	5/161 (3.1)	2/224 (0.9)	2.26 (0.40–12.70)	3.96 (0.61–25.64)
Parechovirus/Enterovirus	19/293 (6.5)	12/161 (7.5)	19/224 (8.5)	1.05 (0.49–2.23)	1.32 (0.54–3.19)
Human rhinovirus	64/293 (21.8)	30/161 (18.6)	50/224 (22.3)	1.37 (0.84–2.22)	0.99 (0.53–1.85)
Respiratory syncytial virus	79/293 (27.0)	51/161 (31.7)	6/224 (2.7)	**15.18 (6.26–36.85**)	**19.03 (7.48–48.41**)

*Conditional odds ratio derived by logistic regression, adjusting age (in months) and presence of all other pathogens: 2 analyses were combined in the output of this Table: the first with no threshold applied for human cytomegalovirus, *H. influenzae*, *P. jirovecii*, and *S. pneumoniae*, and the second with threshold density cutoffs (as noted below) applied to these pathogens. The first analysis output was used to report the adjusted conditional odds for cytomegalovirus, *H. influenzae*, *P. jirovecii*, and *S. pneumoniae* with no threshold density cutoff applied. The second analysis output was used to report the adjusted conditional odds for all pathogens named in the Table.

†Cutoff density threshold which best distinguished between cases and controls, derived by receiver operating characteristic analysis using leave-one-out cross-validation.

‡Cutoff density for *H. influenzae* (nontype b, and type b) on NP/OP swabs: 5.9 log_10_ copies/mL.

§Cutoff density for *S. pneumoniae* on NP/OP swabs: 6.9 log_10_ copies/mL.

¶Vaccine-type or nonvaccine-type pneumococcus amongst children with high density NP/OP pneumococcal carriage.

‖Cutoff density for *S. pneumoniae* in whole blood specimens: 2.2 log_10_ copies/mL.

**Cutoff density for *P. jirovecii* on NP/OP swabs: 4.0 log_10_ copies/mL.

††Cutoff density for human cytomegalovirus on NP/OP swabs: 4.9 log_10_ copies/mL.

CI indicates confidence interval; CXR+, radiologically confirmed pneumonia; N/E, no estimate; NP/OP, Nasopharyngeal/oropharyngeal.

Bolded values indicates statistically significant results, in which 95% confidence intervals do not include zero.

**TABLE 3.** Conditional Odds Ratios in the Comparison Between All Cases, Cases with Radiologically Confirmed Pneumonia, and Controls: HIV-unexposed Children

Pathogen	All Cases	CXR+ Cases	Controls	Conditional Odds Ratio (95% CI)*	
				All Cases vs. Controls	CXR+ Cases vs. Controls
Any nonviral pathogen	424/464 (91.4)	229/245 (93.5)	545/579 (94.1)	0.67 (0.41–1.08)	0.89 (0.47–1.66)
Any nonviral pathogen, above cutoff density threshold†	373/464 (80.4)	200/245 (81.6)	469/579 (81.0)	0.96 (0.70–1.31)	1.02 (0.69–1.52)
Bacteria					
*Bordetella pertussis*	10/464 (2.2)	6/245 (2.4)	3/579 (0.5)	3.86 (0.97–15.47)	**6.85 (1.56–30.05**)
*Chlamydophila pneumoniae*	7/464 (1.5)	4/245 (1.6)	16/579 (2.8)	0.67 (0.23–1.93)	0.47 (0.11–1.96)
*Haemophilus influenzae* type b	6/464 (1.3)	2/245 (0.8)	5/579 (0.9)	1.48 (0.39–5.57)	0.97 (0.14–6.62)
*Haemophilus influenzae* type b ≥ threshold density‡	3/464 (0.6)	2/245 (0.8)	2/579 (0.3)	1.63 (0.99–2.68)	1.45 (0.76–2.74)
Nontype b *Haemophilus influenzae*	242/464 (52.2)	143/245 (58.4)	267/579 (46.1)	1.21 (0.89–1.64)	**1.56 (1.06–2.30**)
Nontype b *Haemophilus influenzae* ≥ threshold density‡	126/464 (27.2)	80/245 (32.7)	121/579 (20.9)	1.29 (0.92–1.82)	**1.60 (1.06–2.43**)
*Moraxella catarrhalis*	275/464 (59.3)	149/245 (60.8)	384/579 (66.3)	0.77 (0.57–1.04)	0.88 (0.59–1.29)
*Mycoplasma pneumoniae*	4/460 (0.9)	1/243 (0.4)	3/579 (0.5)	1.63 (0.29–9.21)	1.72 (0.14–21.08)
*Streptococcus pneumoniae*	296/464 (63.8)	156/245 (63.7)	399/579 (68.9)	0.91 (0.66–1.25)	0.81 (0.54–1.22)
*Streptococcus pneumoniae* ≥ threshold density§	53/464 (11.4)	33/245 (13.5)	54/579 (9.3)	0.92 (0.56–1.50)	0.98 (0.54–1.77)
Vaccine-type *Streptococcus pneumoniae*¶	14/464 (3.0)	8/245 (3.3)	21/582 (3.6)	0.71 (0.31–1.65)	0.76 (0.27–2.12)
Nonvaccine-type *Streptococcus pneumoniae*¶	37/464 (8.0)	25/245 (10.2)	35/582 (6.0)	0.91 (0.51–1.62)	1.04 (0.53–2.05)
*Streptococcus pneumoniae* in whole blood	33/464 (7.1)	22/245 (9.0)	64/583 (11.0)	0.67 (0.40–1.11)	0.92 (0.50–1.68)
*Streptococcus pneumoniae* in whole blood ≥ threshold density‖	24/464 (5.2)	16/245 (6.5)	31/583 (5.3)	1.08 (0.57–2.04)	1.48 (0.69–3.17)
Salmonella spp	0/464 (0.0)	0/245 (0.0)	0/579 (0.0)	N/E	N/E
*Staphylococcus aureus*	114/464 (24.6)	55/245 (22.4)	102/579 (17.6)	1.41 (0.99–2.02)	1.20 (0.76–1.91)
Fungal species					
*Pneumocystis jirovecii*	58/464 (12.5)	33/245 (13.5)	65/579 (11.2)	1.12 (0.72–1.75)	1.28 (0.73–2.23)
*Pneumocystis jirovecii* ≥ threshold density**	30/464 (6.5)	15/245 (6.1)	19/579 (3.3)	**2.35 (1.22–4.52**)	**2.32 (1.03–5.22**)
Viruses					
Any viral pathogen	397/464 (85.6)	219/245 (89.4)	440/579 (76.0)	**1.86 (1.34–2.58**)	**2.61 (1.66–4.10**)
Any viral pathogen, above cutoff density threshold †	381/464 (82.1)	213/245 (86.9)	406/579 (70.1)	**1.89 (1.40–2.55**)	**2.71 (1.79–4.11**)
Adenovirus	39/461 (8.5)	19/244 (7.8)	46/579 (7.9)	1.63 (0.99–2.68)	1.45 (0.76–2.74)
Human cytomegalovirus	181/461 (39.3)	98/244 (40.2)	294/579 (50.8)	0.70 (0.52–0.94)	0.80 (0.55–1.18)
Human cytomegalovirus ≥ threshold density††	96/461 (20.8)	57/244 (23.4)	174/579 (30.1)	0.58 (0.42–0.81)	0.79 (0.52–1.20)
Coronavirus 229	3/461 (0.7)	1/244 (0.4)	1/579 (0.2)	4.86 (0.35–67.39)	3.20 (0.15–68.19)
Coronavirus 43	15/461 (3.3)	7/244 (2.9)	27/579 (4.7)	0.60 (0.28–1.28)	0.77 (0.29–2.04)
Coronavirus 63	12/461 (2.6)	5/244 (2.0)	22/579 (3.8)	1.25 (0.58–2.71)	1.02 (0.35–2.96)
Coronavirus HKU	3/461 (0.7)	3/244 (1.2)	14/579 (2.4)	0.42 (0.11–1.57)	0.96 (0.25–3.66)
Influenza A	14/461 (3.0)	8/244 (3.3)	10/579 (1.7)	**3.72 (1.52–9.11**)	**4.13 (1.45–11.73**)
Influenza B	7/461 (1.5)	5/244 (2.0)	2/579 (0.3)	**5.55 (1.03–30.02**)	**9.07 (1.49–55.26**)
Influenza C	4/464 (0.9)	3/245 (1.2)	6/579 (1.0)	1.06 (0.26–4.36)	1.81 (0.33–9.87)
Human bocavirus	52/460 (11.3)	27/243 (11.1)	60/579 (10.4)	1.19 (0.75–1.89)	1.45 (0.80–2.60)
Human metapneumovirus A/B	28/460 (6.1)	16/243 (6.6)	19/579 (3.3)	1.63 (0.99–2.68)	1.45 (0.76–2.74)
Parainfluenza virus 1	10/460 (2.2)	8/243 (3.3)	2/579 (0.3)	**11.45 (2.42–54.17**)	**19.15 (3.84–95.52**)
Parainfluenza virus 2	1/461 (0.2)	1/244 (0.4)	6/579 (1.0)	0.27 (0.03–2.36)	0.64 (0.07–5.73)
Parainfluenza virus 3	31/461 (6.7)	19/244 (7.8)	11/579 (1.9)	**5.49 (2.63–11.46**)	**6.90 (3.03–15.69**)
Parainfluenza virus 4	9/461 (2.0)	4/244 (1.6)	10/579 (1.7)	1.47 (0.53–4.07)	1.40 (0.37–5.31)
Parechovirus/Enterovirus	27/461 (5.9)	11/244 (4.5)	42/579 (7.3)	1.10 (0.63–1.93)	0.63 (0.28–1.42)
Human rhinovirus	101/461 (21.9)	49/244 (20.1)	133/579 (23.0)	1.35 (0.96–1.89)	1.20 (0.77–1.86)
Respiratory syncytial virus	138/461 (29.9)	84/244 (34.4)	21/579 (3.6)	**13.43 (8.13–22.17**)	**18.04 (10.45–31.17**)

*Conditional odds ratio derived by logistic regression, adjusting age (in months) and presence of all other pathogens: 2 analyses were combined in the output of this Table: the first with no threshold applied for human cytomegalovirus, *H. influenzae*, *P. jirovecii*, and *S. pneumoniae*, and the second with threshold density cutoffs (as noted below) applied to these pathogens. The first analysis output was used to report the adjusted conditional odds for cytomegalovirus, *H. influenzae*, *P. jirovecii*, and *S. pneumoniae* with no threshold density cutoff applied. The second analysis output was used to report the adjusted conditional odds for all pathogens named in the Table.

†Cutoff density threshold which best distinguished between cases and controls, derived by receiver operating characteristic analysis using leave-one-out cross-validation.

‡Cutoff density for *H. influenzae* (nontype b, and type b) on NP/OP swabs: 5.9 log_10_ copies/mL.

§Cutoff density for *S. pneumoniae* on NP/OP swabs: 6.9 log_10_ copies/mL.

¶Vaccine-type pneumococcus amongst children with high density NP/OP pneumococcal carriage.

‖Cutoff density for *S. pneumoniae* in whole blood specimens: 2.2 log_10_ copies/mL.

**Cutoff density for *P. jirovecii* on NP/OP swabs: 4.0 log_10_ copies/mL.

††Cutoff density for human cytomegalovirus on NP/OP swabs: 4.9 log_10_ copies/mL.

CI indicates confidence interval; CXR+, radiologically confirmed pneumonia; N/E, no estimate; NP/OP, Nasopharyngeal/oropharyngeal.

Bolded values indicates statistically significant results, in which 95% confidence intervals do not include zero.

In addition to the pathogens listed above in the HIV-exposure status stratified analyses, human metapneumovirus (HMPV: aOR, 3.39; 95% CI, 1.88–6.13), adenovirus (aOR, 1.55; 95% CI, 1.01–2.39) and high-density Hib (aOR, 1.55; 95% CI, 1.01–2.39) NP/OP carriage were associated with radiologically confirmed pneumonia case-status in HIV-uninfected children as a combined group (Supplemental Digital Content 7, http://links.lww.com/INF/D835).

Among the eight HIV-exposed children who died in hospital, high-density NP/OP carriage of P. *jirovecii* and HMPV were significantly higher (both 2/8 cases each) compared to control carriage (Supplemental Digital Content 8, http://links.lww.com/INF/D836). Among the 11 HIV-unexposed children who died in hospital, parainfluenza virus 1, *B. pertussis*, Hib, high-density *P. jirovecii* and adenovirus were significantly more prevalent compared to HIV-unexposed community controls (Supplemental Digital Content 9, http://links.lww.com/INF/D837). Among all HIV-uninfected children combined, RSV was more prevalent among those who died (2/20, 10.0%) compared to controls (27/823, 3.3%), but did not reach significance (aOR 5.5, 95% CI 0.98–31.3), while a significantly lower prevalence of *Moraxella catarrhalis* NP/OP carriage was noted in HIV-uninfected children who died (7/20, 35.0%), compared with community controls (548/823, 66.6%) (aOR 0.20; 95% CI, 0.06–0.65) (Supplemental Digital Content 10, http://links.lww.com/INF/D838).

### PERCH Integrated Analysis Determination of Pathogen Etiology Fraction in HIV-uninfected South African Children, Stratified by HIV-exposure Status

**FIGURE 1.**
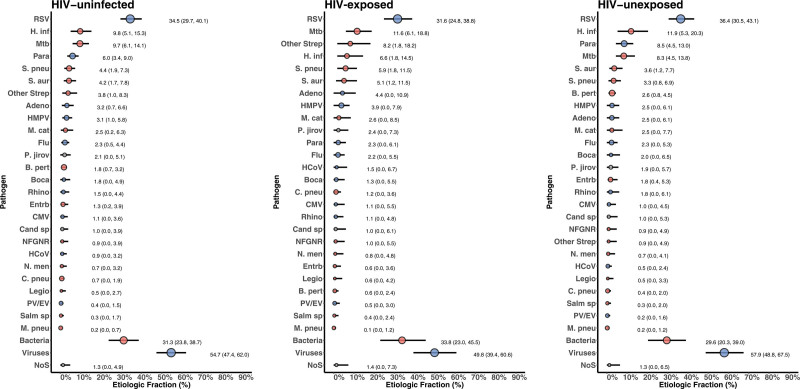
Integrated etiology results for HIV-exposed and HIV-unexposed cases with radiologically confirmed pneumonia. Sample size: N = 165 (HIV-exposed); N = 246 (HIV-unexposed). *C. pneu, Chlamydophila pneumoniae*; *Cand* sp, *Candida* species; Entrb, Enterobacteriaceae; Flu, Influenza virus A, B and C; HCoV, human coronavirus; *Legio*, Legionella species; NFGNR, nonfermentative Gram-negative rods; *N. men*, *Neisseria meningitidis*; NoS, not otherwise specified (ie, pathogens not tested for); *P. jirov, Pneumocystis jirovecii*; PV/EV, parechovirus/enterovirus; Salm sp, Salmonella species. Other Strep includes *Streptococcus pyogenes* and *Enterococcus faecium*. NFGNR includes Acinetobacter species and Pseudomonas species. Enterobacteriaceae includes *E. coli*, Enterobacter species, and Klebsiella species, excluding mixed Gram-negative rods. Radiologically confirmed defined as consolidation and/or other infiltrate on chest radiograph. Bacterial summary excludes *Mtb*. Pathogens estimated at the subspecies level but grouped to the species level for display (Parainfluenza virus type 1, 2, 3 and 4; *S. pneumoniae* PCV13 and *S. pneumoniae* non-PCV13 types; *H. influenzae* type b and *H. influenzae* non-b; influenza A, B, and C). Estimates for subspecies and serotype disaggregation (eg, PCV13 type and non-PCV13 type), are given in Table 4 (age-stratified analysis) and Supplemental Digital Content 13, http://links.lww.com/INF/D841 (pneumonia severity-stratified analysis) for the top 10 pathogens. Description of symbols: Line represents the 95% credible interval. The size of the symbol is scaled based on the ratio of the estimated etiologic fraction to its SE. Of 2 identical etiologic fraction estimates, the estimate associated with a larger symbol is more informed by the data than the priors.

Bayesian analytic outputs identified RSV as being the most important contributor to severe/very severe pneumonia in HIV-exposed (EF 31.6%) and HIV-unexposed (EF 36.4%) children hospitalized with radiologically confirmed pneumonia (Fig. 1). Furthermore, *Mtb* contributed substantially to the EF of radiologically confirmed pneumonia in HIV-exposed (EF 11.6%) and HIV-unexposed (EF 8.3%) children (Fig. 1). Overall, respiratory viral pathogens contributed a larger combined EF in HIV-exposed and HIV-unexposed children than did bacteria (Fig. 1).

**TABLE 4.** Top 10 Pathogens Associated With Radiologically Confirmed Pneumonia in HIV-uninfected Children, Stratified HIV-exposure Status and Age

	HIV-uninfected				HIV-exposed Children				HIV-unexposed Children			
	Age 1–11 months		Age 12–59 months		Age 1–11 months		Age 12–59 months		Age 1–11 months		Age 12–59 months	
	Pathogen	EF (95% CrI)	Pathogen	EF (95% CrI)	Pathogen	EF (95% CrI)	Pathogen	EF (95% CrI)	Pathogen	EF (95% CrI)	Pathogen	EF (95% CrI)
1	RSV	43.0 (37.4–49.7)	*Hi* non-b	22.2 (8.9–37.6)	RSV	38.2 (30.3–47.0)	*Mtb*	16.3 (6.1–33.3)	RSV	46.5 (40.4–54.5)	*Hi* non-b	29.1 (10.3–50.0)
2	*Mtb*	10.0 (5.8–15.5)	*Mtb*	8.6 (3.0–16.8)	*Mtb*	10.4 (4.5–18.9)	*S. pneu* PCV13	12.3 (0.0–27.3)	*Mtb*	9.7 (5.1–16.3)	RSV	10.1 (0.0–22.1)
3	Para	6.0 (2.9–9.4)	RSV	8.4 (0.0–16.8)	Other Strep	9.8 (2.3–22.0)	*Hi* non-b	7.9 (0.0–30.3)	Para	9.1 (4.5–14.0)	Para	7.0 (0.0–22.1)
4	*S. aur*	4.6 (1.6–8.7)	Para	6.0 (1.0–12.9)	*S. aur*	5.2 (0.8–12.9)	*M. cat*	5.6 (0.0–24.2)	*Hi* non-b	4.6 (0.0–12.9)	HBOV	5.7 (0.0–22.1)
5	Other Strep	4.5 (1.0–9.7)	*M. cat*	5.1 (0.0–16.8)	Adeno	4.2 (0.0–12.1)	Adeno	5.2 (0.0–18.2)	*S. aur*	4.1 (1.1–8.4)	Rhino	5.1 (0.0–19.1)
6	*Hi* non-b	4.2 (0.6–9.7)	Adeno	5.1 (0.0–13.9)	HMPV	3.8 (0.0–8.3)	RSV	4.9 (0.0–15.2)	*B. pert*	3.5 (1.1–6.2)	Adeno	5.0 (0.0–16.2)
7	HMPV	3.1 (0.6–6.1)	*S. pneu* PCV13	5.0 (1.0–9.9)	*Hi* non-b	3.6 (0.0–11.4)	*S. aur*	4.8 (3.0–12.1)	*S. pneu* Non-PCV13	2.7 (0.0–7.3)	*M. cat*	4.9 (0.0–20.6)
8	*P. jirov*	2.8 (0.0–6.8)	HBOV	4.4 (0.0–15.8)	*P. jirov*	2.9 (0.0–9.1)	*S. pneu* Non-PCV13	4.2 (0.0–15.2)	*P. jirov*	2.6 (0.0–7.9)	*Mtb*	4.9 (1.5–14.7)
9	Adeno	2.6 (0.3–6.5)	Rhino	4.0 (0.0–13.9)	*S. pneu* PCV13	2.9 (0.0–8.3)	HMPV	4.2 (0.0–15.2)	HMPV	2.6 (0.0–6.7)	Flu	3.3 (0.0–10.3)
10	*B. pert*	2.3 (0.6–4.2)	*S. aur*	3.2 (1.0–9.9)	Flu	2.0 (0.0–5.3)	Para	3.9 (0.0–12.1)	Entrb	2.2 (0.6–6.7)	*S. aur*	2.5 (0.0–11.8)
	**Top 10**	**83.1 (75.5–89.7**)	**Top 10**	**72.2 (55.4–86.1**)	**Top 10**	**83.1 (70.5–93.2**)	**Top 10**	**69.3 (45.5–90.9**)	**Top 10**	**87.6 (78.7–94.9**)	**Top 10**	**77.6 (55.9–92.6**)

Entrb indicates Enterobacteriaceae; Flu, influenza virus.

Other Strep includes *Streptococcus pyogenes* and *Enterococcus faecium*. Enterobacteriaceae includes *E. coli*, Enterobacter species, and Klebsiella species, excluding mixed Gram-negative rods.

Radiologically confirmed defined as consolidation and/or other infiltrate on chest radiograph. Bolded values indicates statistically significant results, in which 95% confidence intervals do not include zero.

### Age- and Pneumonia Severity-stratified Analysis for Etiologic Fraction Estimation in HIV-exposed and HIV-unexposed Children

The “top 10” pathogens associated with radiologically confirmed pneumonia in HIV-exposed and HIV-unexposed children, stratified by age group, are shown in Table 4. RSV and *Mtb* were the first and second ranked pathogens associated with radiologically confirmed pneumonia in children <12 months of age in HIV-exposed (EF 38.2% and 10.4%) and HIV-unexposed (EF 46.5% and 9.7%) children (Table 4). Pneumococcus and *Mtb* were the first and second ranked pathogens (EF 16.5% and 16.3%) associated with radiologically confirmed pneumonia in HIV-exposed children ≥12 months of age (Supplemental Digital Content 11, http://links.lww.com/INF/D839 and Table 4). The “top 10” organisms contributed over 69% of the EF in each age group (Table 4), and the contribution of the “not otherwise specified” category to pneumonia etiology was 2.5% or less (Supplemental Digital Content 11, http://links.lww.com/INF/D839 and Supplemental Digital Content 12, http://links.lww.com/INF/D840).

When stratifying by pneumonia severity, RSV remained the top-ranked pathogen in HIV-uninfected children overall, HIV-exposed and HIV-unexposed children (Supplemental Digital Content 13, http://links.lww.com/INF/D841). Furthermore, in the severity-stratified analysis, *Mtb* was consistently implicated within the “top 10” ranked organisms regardless of HIV-exposure status. *P. jirovecii* ranked fifth in association with severe pneumonia in HIV-exposed children (EF 3.5%; 95% CrI, 0.0%–9.9%) but did not feature in the “top 10” organisms associated with pneumonia in HIV-unexposed children (Supplemental Digital Content 13, http://links.lww.com/INF/D841).

Vaccine-type pneumococci contributed to the “top 10” organisms associated with radiologically confirmed pneumonia in the age- (EF 2.9%, <12 months; EF 12.3%, ≥12 months) and severity-stratified (EF 3.2%, severe; EF 10.0%, very severe) analyses amongst HIV-exposed children. In contrast, nonvaccine-type pneumococci (EF 2.7%, <12 months; EF 4.8%, very severe), but not vaccine-type pneumococci, contributed to the “top 10” organisms associated with radiologically confirmed pneumonia in HIV-unexposed children (Table 4 and Supplemental Digital Content 13, http://links.lww.com/INF/D841).

### Sensitivity Analysis in the PERCH Integrated Analysis Outputs for *Mtb* in the South African PERCH Cohort

Sensitivity analyses which adopted a lower (10%–30%) sensitivity prior for *Mtb* culture from clinical specimens resulted in higher EFs attributable to *Mtb* in the South African HIV-uninfected cohort, including an EF of 19.5% (95% CrI, 6.1%–36.4%) in HIV-exposed children >12 months of age (compare Table 4, Supplemental Digital Content 13, http://links.lww.com/INF/D841 and Supplemental Digital Content 14, http://links.lww.com/INF/D842).

## DISCUSSION

The South African site contributed 25% (435/1769) of HIV-uninfected cases with radiologically confirmed pneumonia enrolled in the multi-site PERCH study.^34^ The results of the analyses presented here give further insight into the etiology of childhood pneumonia in a low-middle income sub-Saharan African setting with established vaccination programmes against Hib and pneumococcus as well as a high HIV- and tuberculosis burden. In summary analysis, illness severity and age-stratified analyses (amongst infants), RSV was implicated as the leading pathogen associated with radiologically confirmed pneumonia in HIV-uninfected South African children, as also observed across all other PERCH sites.^34^ RSV has also been identified as the leading cause of community-acquired pneumonia (CAP) in other recent studies.^6,35,36^

Furthermore, PIA outputs for HIV-uninfected children consistently highlighted the prominent role of *Mtb* in CAP etiology in the South Africa African HIV-uninfected children, despite high Bacillus Calmette-Guérin coverage in the cohort. *Mtb* featured within in the “top 10” organisms associated with childhood severe/very severe pneumonia at all PERCH sites,^34^ 3 of which (South Africa, Kenya and Zambia) are classified among the 22 high-burdened settings of tuberculosis. At the time of PERCH study conduct (2011, through 2013) the tuberculosis incidence rate in South Africa declined from 922 per 100,000 to 849 per 100,000.^37^ Marked improvements in integration of HIV and tuberculosis services in South Africa, with expedited access onto antiretroviral treatment for HIV-infected persons, has driven this decline in tuberculosis incidence.^38^ Documented significant reductions in childhood microbiologically confirmed pulmonary tuberculosis in South Africa have been noted in HIV-infected and -uninfected children, also reflecting up-scaled access to antiretroviral treatment for HIV-infected individuals.^39,40^

The cumulative incidence (per 100,000 population) of culture-confirmed pulmonary tuberculosis in HIV-uninfected Sowetan children under 5 years of age in 2012, the midpoint of PERCH enrollment activities at the South African site, was 27.5 (95% CI, 18.8–38.8),^40^ which is similar to the current WHO estimate for microbiologically confirmed childhood tuberculosis in South Africa (39; 95% CI, 25–52).^41^ When taking into consideration the imperfect sensitivity (20%–50% in the current analyses) of culture-confirmation of disease caused by *Mtb*, these incidence rates likely represent a conservative estimate of the role of *Mtb* in acute severe/very severe pneumonia in our setting.

HIV-exposed status appears to be an important determinant of *Mtb*-associated CAP in South Africa, as evidenced by the PIA outputs which ranked the organism first amongst HIV-exposed children ≥12 months of age (Table 2, Supplemental Digital Content 1, http://links.lww.com/INF/D829). Such a finding is biologically feasible, in that preschool aged children residing in households of HIV-infected caregivers have been shown to be at-risk for *Mtb* infection.^42,43^ High rates of exposure to infectious tuberculosis cases perpetuate the burden of latent infection and disease in their close contacts, which is concerning considering that coverage of isoniazid preventive therapy for at-risk children in South Africa is suboptimal,^44–46^ despite compelling evidence of its effectiveness in preventing tuberculosis.^47^

Relative immune paresis of HIV-exposed infants, compared to those that are HIV-unexposed,^48^ may explain the contribution of *P. jirovecii* and vaccine-serotype pneumococci to case-status in HIV-exposed children in this study. Although *P. jirovecii* contributed similar EFs (2.4% vs. 1.9%) to pneumonia etiology in HIV-exposed and HIV-unexposed children in the South African PERCH cohort, it was associated with disease in the youngest children (regardless of HIV-exposure status) and featured among the “top 10” pathogens associated with severe pneumonia in HIV-exposed infants in severity-stratified analyses. *P. jirovecii* was also significantly associated with case-status among children dying in-hospital. Heightened risk for disease associated with vaccine preventable infections amongst HIV-exposed children was evidenced by the contribution of vaccine-serotype pneumococci amongst the “top 10” pneumonia pathogens in HIV-exposed children at the South African PERCH site. In contrast, nonvaccine serotype pneumococci were implicated among the “top 10” pathogens in HIV-unexposed children. High-density vaccine-type pneumococcal carriage prevalence in cases with radiologically-confirmed pneumonia were 6.1% (10/164) and 3.3% (8/245) in HIV-exposed and HIV-unexposed children, respectively. Certain studies have demonstrated less robust responses to PCV in HIV-exposed compared to HIV-unexposed children.^49^

The wider spectrum of nonviral pathogens (*B. pertussis*, *H. influenzae* and *P. jirovecii*) that were statistically associated with case-status in the conditional logistic regression analyses of NP/OP carriage in HIV-unexposed compared with HIV-exposed children could represent a type-I sampling error, incurred by smaller numbers of HIV-exposed than HIV-unexposed children in this study. However, some pathogens were more prevalent amongst HIV-unexposed than amongst HIV-exposed cases with radiologically-confirmed pneumonia, such as parainfluenza 1 (3.3% vs. 0.6%) and parainfluenza 3 (7.8% vs. 3.7%). Cohen et al^6^ reported significantly higher incidence rates of lower respiratory tract illness associated with adenovirus, enterovirus, HMPV, human rhinovirus, parainfluenza virus 1 and RSV in HIV-exposed compared with HIV-unexposed South African children, albeit in a cohort of infants with respiratory illness requiring hospitalization but not confined to WHO severe/very severe pneumonia as was the case in PERCH.

Viral pathogens were associated with pneumonia etiology in 49.8% (95% CrI, 39.4%–60.6%) HIV-exposed children and 57.9% (95% CrI, 48.8%–67.5%) HIV-unexposed children with radiologically confirmed pneumonia, which contrasts with the 26.8% (95% CrI, 15.7%–38.2%) viral etiologic estimate in HIV-infected South African children.^13^ This may have implications for empiric therapy of CAP in HIV-uninfected children residing in regions with widespread vaccine coverage against Hib and pneumococcus if the clinical scenario permits, so as to limit the use of empiric antibiotic therapy through clinical guidelines either encouraging avoidance of antibiotic therapy or promoting short-course, narrow-spectrum antibiotic therapy. Few respiratory viral pathogens are preventable through vaccination, and a limited armamentarium of antiviral agents are available to treat severe disease attributable to these pathogens. Uptake of influenza vaccination in sub-Saharan Africa is limited, and vaccine effectiveness is unpredictable.^50^ A fresh realization of the contribution that RSV makes to the global pediatric pneumonia burden has invigorated efforts to develop safe and efficacious RSV vaccines.^51^ A strategy of vaccinating pregnant women against influenza has been shown to impact considerably on the burden of all-cause pneumonia hospitalizations among their infants.^52^ A recent trial of antenatal RSV vaccine administration has shown efficacy in preventing severe RSV-associated pneumonia in infants.^53^

## CONCLUSIONS

RSV contributes substantially to the burden of severe/very severe pneumonia requiring hospitalization in HIV-uninfected children in South Africa. A safe, effective vaccine against RSV would be anticipated to impact substantially on the burden of pneumonia hospitalization amongst young children in this setting. Furthermore, *Mtb* was prominently associated with HIV-uninfected case-status in all age, severity, and HIV-exposure groups. Efforts must be made to strengthen tuberculosis programmes in South Africa, including a renewed emphasis on the importance of isoniazid preventive therapy in child contacts of infectious tuberculosis cases, as well as active case finding so as to identify undiagnosed close contacts of newly diagnosed tuberculosis patients.

## ACKNOWLEDGMENTS

The authors are grateful for the participation of all of the children and their families who participated in PERCH at the South African site. Substantial input with regards site-specific study and laboratory set-up were made by Michelle J. Groome and Peter V. Adrian at the Respiratory & Meningeal Pathogens Research Unit at Chris Hani Baragwanath Academic Hospital. Substantial oversight of PERCH activities was made by Amanda J. Driscoll through the Department of International Health, International Vaccine Access Center, Johns Hopkins Bloomberg School of Public Health, Baltimore, MD. Socio-economic stratification of PERCH participants was derived through analyses conducted by Elizabeth Chmielewski-Yee. Data quality assurance was provided by Nora L. Watson at The Emmes Corporation, Rockville, MD, and the Bayesian analysis was undertaken by Zhenke Wu and Scott L. Zeger at the Department of Biostatistics, Johns Hopkins University, Baltimore, MD.

## Supplementary Material


