# Supplementary material for: The Etiology of Pneumonia in HIV-uninfected South African Children: Findings From the Pneumonia Etiology Research for Child Health (PERCH) Study
Source: Pediatr Infect Dis J. 2021 Aug 25;40(9):S59–68. doi: 10.1097/INF.0000000000002650 (PMC8448398; doi:10.1097/INF.0000000000002650)
Supplement: Supplementary file 1 [file inf-40-s59-s001.docx]

# SUPPLEMENTAL DIGITAL CONTENT 1

Information contained in Supplemental Digital Content 1 elaborates on the South African Pneumonia Etiology Research for Child Health (PERCH) catchment area and aspects of the study methodology, as it pertained to HIV-uninfected children.

## Study Catchment Area

The study catchment area, situated in Regions D and G or the City of Johannesburg, in the Gauteng Province of South Africa, was defined as the region from which 90% of PERCH age-eligible pediatric admissions to Chris Hani Baragwanath Academic Hospital (CHBAH) resided in 2010, the year preceding commencement of PERCH enrolment. The catchment area had a population of 1.5 million in 2011, approximately 150,000 of which were children under-5 years of age. In 2011, the average population density was 5,208 people/km^2^, and 64% of households were classified as formal structures. The unemployment rate in 2011 was 32%, and 20% of all households had no fixed income.^(1)^ Official estimates of third dose *Haemophilus influenzae* type b conjugate vaccine (HibCV) and pneumococcal conjugate vaccine (PCV) coverage in South Africa were 83% and 88% in 2012.^(2)^

## Community Control Selection Procedure

Potential community controls were identified by reviewing hospital birth registers using randomized lists of birth dates which corresponded to the age categories (1-5 months, 6-11 months, 12-23 months and 24-59 months) of enrolled cases. These lists were compiled each month during study conduct, and birth registers were reviewed weekly. Names of potential controls, and their addresses, were abstracted from birth registers, and were compiled into lists from which community control recruitment visits could be conducted.

Field workers visited the households of potential controls on weekdays to invite them to participate in the study. If the pre-selected, age-frequency matched potential control was not found at his/her address on the day of the field visit, the field worker visited each consecutive neighboring household until an alternative age-matched control was identified. The maximum time frame used to source an alternative community control by going from house-to-house, was 30 minutes.

The parents/legal guardians of potential controls were advised to accompany the child to the study clinic at CHBAH within two working days, and no later than a week, of the field worker’s visit. Study clinic hours were from 08:00 until 16:00 on weekdays.

## Specimen Collection from HIV-uninfected Cases and Controls

Routine investigations done on cases included blood culture, complete blood count, C-reactive protein (CRP), gastric aspirates (GA) for mycobacterial culture, and chest radiographs, as previously described.^(3-6)^ Study-specific investigations included collection of nasopharyngeal (NP) and oropharyngeal (OP) swabs for multiplex polymerase chain reaction (PCR) testing (Fast Track Diagnostics Respiratory Pathogens 33 test (FTD-33), Fast Track Diagnostics, Sliema, Malta).^(7-9)^ Cases at the South African PERCH site also underwent collection of two induced sputum (IS) specimens for routine microbiology, mycobacterial culture and FTD-33 PCR testing.^(5)^ Similarly, mechanically ventilated children had endotracheal tracheal (ETT) aspirates submitted for microbiological culture, mycobacterial culture, and FTD-33 PCR testing. Cases with pleural effusions, and a limited number who had peripherally-located consolidation on chest radiograph and whose parents permitted lung aspirate (LA) sampling, had pleural fluid or LA fluid submitted for routine microbiological culture, mycobacterial culture, and FTD-33 PCR testing.^(10)^

Controls had nasopharyngeal-oropharyngeal (NP/OP) swabs collected in universal transport medium, for FTD-33 PCR testing.^(10)^ Blood was collected from all controls for hemoglobin, and a subset had their blood tested for CRP.^(4)^

Serum specimens were collected from cases and controls, for determination of antibiotic activity at the time of enrollment into PERCH.^(11)^ Cases and controls also had blood samples collected for *lytA* PCR,^(12)^ and HIV infection status testing using age- and HIV-exposure status appropriate assays. Children <18 months of age with a history of HIV-exposure, or a positive HIV serologic test, were tested using qualitative HIV PCR (Roche Amplicor HIV DNA PCR assay version 1.5). Children ≥18 months of age, or those <18 months but with no history of HIV-exposure, were tested using enzyme-linked immunosorbent assay (ELISA) or rapid HIV tests.

## Determination of HIV-exposure Status

HIV-exposure status was determined using a cluster of parameters, including: age of the participant, his/her HIV serostatus (in those <18 months of age), and maternal HIV history and/or her serostatus at PERCH enrolment.

Children were deemed to be HIV-exposed, -uninfected if maternal HIV-status was reported to be positive during pregnancy and age-appropriate HIV test results on the child were negative, or if a serologic HIV test was positive, but HIV PCR negative in children <18 months of age in those whose mothers did not report being HIV-infected during pregnancy. Children <7 months of age were deemed to be HIV-unexposed if they had a negative serologic HIV test. In older children, HIV-unexposed status was assigned if there was documented negative maternal serostatus during pregnancy, post-delivery or at PERCH enrolment. Children ≥7 months of age with negative serologic tests for HIV, and maternal history of being HIV-uninfected were presumed to be unexposed, and were included in the HIV-unexposed category. Children with unknown maternal HIV status and missing HIV serology results were classified as having unknown HIV-exposure status.

***The PERCH Integrated Analysis***

The percent of pneumonia due to each pathogen was estimated using the PERCH Integrated Analysis (PIA) method, which is described in detail elsewhere.^(13-15)^ In brief, the PIA is a Bayesian nested partially latent class analysis that integrates the results for each case from blood culture, NP/OP PCR, whole blood PCR for pneumococcus and induced sputum culture for *Mycobacterium tuberculosis* (*Mtb*). The PIA also integrates test results from controls to account for imperfect test specificity of NP/OP PCR and whole blood PCR. Blood culture results (excluding contaminants) and *Mtb* results were assumed to be 100% specific.

The PIA accounts for imperfect sensitivity of each test/pathogen measurement by using *a priori* estimates of their sensitivity (i.e., estimates regarding the plausibility range of sensitivity which varied by laboratory test method and pathogen). Sensitivity of blood culture was reduced if blood volume was low (<1.5 mL) or if antibiotics were administered before specimen collection. Sensitivity of NP/OP PCR for *Streptococcus pneumoniae* and *Haemophilus influenzae* was reduced if antibiotics were administered before specimen collection (Supplementary Digital Content 1 (SDC-1) Table 1).

As a Bayesian analysis, both the list of pathogens and their starting ‘prior’ etiologic fraction values were specified *a priori,* which favored no pathogen over another (i.e., ‘uniform’). The pathogens selected for inclusion in the analysis included any non-contaminant bacteria detected by culture in blood at any of the 9 PERCH sites, regardless of whether it was observed at the South African site specifically, *Mtb* and all of the multiplex quantitative PCR pathogens except those considered invalid because of poor assay specificity (*Klebsiella* *pneumoniae*^(16)^ and *Moraxella catarrhalis*).

**SDC-1 Table 1: Integrated etiology analysis input values for sensitivity and specificity of laboratory test measures**

|  |  | **Sensitivity Prior^a^** | |  |
| --- | --- | --- | --- | --- |
| **Specimen/test** | **Pathogen** | **Base** | **Reduced** | **Specificity** |
| Blood cultures^c^ | *Streptococcus pneumoniae*  *Haemophilus influenzae* | 5-20% | 1-13% | 100% |
|  | *Moraxella catarrhalis*  *Staphylococcus aureus*  Non-fermentative Gram-negative rods  Candida species  Non-pneumococcal streptococci, including enterococci | 5-15% | 1-10% |  |
|  | Salmonella species  Enterobacteriaceae  *Neisseria meningitidis* | 10-50% | 1-34% |  |
| NP/OP PCR | *Streptococcus pneumoniae*  *Haemophilus influenzae* | 50-90% | 15-55% | 1 − Control prevalence (ref Table 2, 3 and SDC 7) |
|  | Salmonella species  Legionella species | 0.5-90% | 0.5-90% |  |
|  | All other PCR targets | 50-90% | 50-90% |  |
| Whole blood PCR | *Streptococcus pneumoniae* | 12-65% | 12-65% | 1 − Control prevalence (ref Table 2, 3 and SDC 7) |
| Induced sputum | *Mycobacterium tuberculosis* | 20-50% | 20-50% | 100% |

Abbreviations: SDC = Supplementary Digital Content

a. Background information supporting choice of sensitivity priors provided in the all-site PERCH paper.^(15)^

b. Base: >1.5 mL blood culture volume (blood culture only) and no evidence of prior antibiotic exposure. Reduced <1.5 mL, or evidence of prior antibiotic exposure.

c. Direct evidence of the diagnostic sensitivity for *Streptococcus pneumoniae* and *Haemophilus influenzae* from vaccine probe studies. For all other pathogens, we set the base blood culture sensitivity prior to 5-15%, with the exception of Salmonella species, Enterobacteriaceae and *Neisseria meningitidis*, for which we selected wider priors (10-50%) to reflect their greater uncertainty.

A category called ‘Pathogens Not Otherwise Specified’ (NoS) was also included to estimate the fraction of pneumonia caused by pathogens not tested for or not observed. A child negative for all pathogens would still be assigned an etiology, which would be either one of the explicitly estimated pathogens (implying a ‘false negative’, accounting for imperfect sensitivity of certain measurements) or NoS.

The PIA estimated both the individual and population-level etiology probability distributions, each summing to 100% across pathogens where each pathogen has a probability ranging from 0% to 100%. The population level etiologic fraction estimate for each pathogen was approximately the average of the individual case probabilities and was provided with a 95% Credible Interval (95% CrI), the Bayesian analogue of the Confidence Interval (CI).

## Tuberculosis in the HIV-uninfected South African PERCH Cohort

*Mycobacterium tuberculosis* featured prominently in the South African PERCH Integrated Analysis (PIA) outputs, contributing an etiologic fraction of 6.4% (95% CrI, 3.1-11.3%) amongst HIV-uninfected children with radiologically-confirmed pneumonia in the overarching PERCH analysis,^(15)^ as well as in sensitivity analyses in the HIV-exposure stratified analysis presented in this site-specific analysis (in which the largest contribution of *Mtb* was 19.5% (95% CrI, 6.1-36.4%) amongst HIV-exposed, uninfected children >12 months of age). A sensitivity prior of 10-30%, which is informed by the literature,^(17)^ was used for *Mtb* in the sensitivity analyses. However, in foundational PERCH analyses, the first induced sputum was estimated to have a sensitivity of 36.0% (95% CI, 18.0-57.5%) in detecting nine of 25 South African children with culture-confirmed pulmonary tuberculosis in whom two induced sputum specimens had been collected.^(5)^ As South African has a high burden of tuberculosis,^(18)^ we made a decision to use a higher sensitivity prior (20-50%) for tuberculosis culture in the site-specific etiology analysis.

SDC-1 Table 2 summarises the number of HIV-uninfected children diagnosed with tuberculosis at the South African PERCH site, and highlights that in children ≥12 months of age, microbiologically-confirmed tuberculosis prevalence was substantially greater in HIV-exposed, -uninfected compared to HIV-unexposed children (7.8% vs. 3.8% for all children; 6.1% vs. 1.5% for children with radiologically-confirmed

**SDC-1 Table 2: Tuberculosis Diagnosed in HIV-uninfected Children, Stratified by HIV-exposure Status and Age**

|  | | HIV-exposed | | | HIV-unexposed | | |
| --- | --- | --- | --- | --- | --- | --- | --- |
|  |  | <12 months | ≥12 months | All | <12 months | ≥12 months | All |
| All Cases | Microbiologically-confirmed TB^a^ | 7/234 (3.0%) | 5/64 (7.8%) | 12/298 (4.0%) | 7/334 (2.1%) | 5/131 (3.8%) | 12/465 (2.6%) |
|  | Clinically diagnosed TB^b^ | 18/234 (7.7%) | 8/64 (12.5%) | 26/298 (8.7%) | 30/333 (9.0%) | 15/131 (11.5%) | 45/464 (9.7%) |
|  | Total | 25/234 (10.7%) | 13/64 (20.3%) | 38/298 (12.8%) | 37/334 (11.1%) | 20/131 (15.3%) | 57/465 (12.3%) |
| CXR+ Cases | Microbiologically-confirmed TB^a^ | 7/132 (5.3%) | 2/33 (6.1%) | 9/165 (5.5%) | 6/178 (3.4%) | 1/68 (1.5%) | 7/246 (2.8%) |
|  | Clinically diagnosed TB^b^ | 12/132 (9.1%) | 6/33 (18.2%) | 18/165 (10.9%) | 14/177 (7.9%) | 7/68 (10.3%) | 21/245 (8.6%) |
|  | Total | 19/132 (14.4%) | 8/33 (24.2%) | 27/165 (16.4%) | 20/178 (11.9%) | 8/68 (11.8%) | 28/246 (11.4%) |

Abbreviations: CXR = Chest radiograph; CXR+ = Radiologically-confirmed pneumonia; HEU = HIV-exposed, -uninfected; HIV = Human immunodeficiency virus type-1; TB = Tuberculosis.

^a^ Microbiologically-confirmed TB: *Mycobacterium tuberculosis* (*Mtb*) cultured from respiratory specimens in children with compatible clinical presentation of tuberculosis.

^b^ Clinically diagnosed TB: Diagnosis of tuberculosis made clinically, and child initiated onto anti-tuberculosis therapy, with no culture-confirmation through isolation of *Mtb* from clinical specimens.

pneumonia). Furthermore, the burden of all tuberculosis cases (both microbiologically-confirmed and clinically diagnosed) was greatest in HIV-exposed, -uninfected children, particularly in those ≥12 months of age.

## Microbiology Results among Cases

In HIV-uninfected children from whom blood culture results were available, there were 17 (2.1%) of 802 cases that isolated clinically significant bacteria indicating the presence of bacteremic pneumonia. Gram negative organisms (n=10) predominated over Gram positive organisms (n=7) in bacteremic pneumonia.

The Gram negative organisms isolated on blood culture included *Escherichia coli* in three HIV-unexposed children, and *H. influenzae* in two female children (*H. influenzae* type b (Hib) in an HIV-exposed 2-month old, and non-type b *H. influenzae* in an HIV-unexposed 13-month old) both of whom had radiologically-confirmed pneumonia.

There were four microbiologically-confirmed pneumococcal cases. Although viable pneumococcus was not cultured on blood, it was identified on latex agglutination testing of a flag-positive, culture negative blood culture in a 5-month old HIV-unexposed female with radiologically-confirmed pneumonia who was up-to-date with PCV doses. Two females, 18 and 35 months old, respectively, who presented with pleural effusions were PCR positive for pneumococcus on pleural fluid testing. A further 19-month old HIV-unexposed male was PCR positive for pneumococcus and *H. influenzae* on LA testing.

Three of the five children subjected to lung aspiration were PCR positive for a variety of pathogens. In addition to the above case with pneumococcal and *H. influenzae* co-infection, there were two others with bacterial-viral co-infections (one with adenovirus and *Chlamydophila pneumoniae*, and the other with human metapneumovirus, *H. influenzae* and *M. catarrhalis*) on LA testing.

## REFERENCES

1. Statistics South Africa. Census 2011 Statistics: City of Johannesburg [Census 2011 Statistics]. 2017. Available at: <http://www.statssa.gov.za/?page_id=993&id=city-of-johannesburg-municipality>. Accessed 29 January 2017.

2. World Health Organization and UNICEF. South Africa: WHO and UNICEF estimates of immunization coverage: 2016 revision. In: World Health Organization and UNICEF, ed. Geneva: World Health Organization and UNICEF; 2016.

3. Driscoll AJ, Karron RA, Morpeth SC, et al. Standardization of Laboratory Methods for the PERCH Study. *Clin Infect Dis*. 2017;64:S245-s252.

4. Higdon MM, Le T, O'Brien KL, et al. Association of C-Reactive Protein With Bacterial and Respiratory Syncytial Virus-Associated Pneumonia Among Children Aged <5 Years in the PERCH Study. *Clin Infect Dis*. 2017;64:S378-s386.

5. Moore DP, Higdon MM, Hammitt LL, et al. The Incremental Value of Repeated Induced Sputum and Gastric Aspirate Samples for the Diagnosis of Pulmonary Tuberculosis in Young Children With Acute Community-Acquired Pneumonia. *Clin Infect Dis*. 2017;64:S309-s316.

6. Fancourt N, Deloria Knoll M, Barger-Kamate B, et al. Standardized Interpretation of Chest Radiographs in Cases of Pediatric Pneumonia From the PERCH Study. *Clin Infect Dis*. 2017;64:S253-s261.

7. Baggett HC, Watson NL, Deloria Knoll M, et al. Density of Upper Respiratory Colonization With *Streptococcus pneumoniae* and Its Role in the Diagnosis of Pneumococcal Pneumonia Among Children Aged <5 Years in the PERCH Study. *Clin Infect Dis*. 2017;64:S317-s327.

8. Park DE, Baggett HC, Howie SRC, et al. Colonization Density of the Upper Respiratory Tract as a Predictor of Pneumonia - *Haemophilus influenzae*, *Moraxella catarrhalis*, *Staphylococcus aureus*, and *Pneumocystis jirovecii*. *Clin Infect Dis*. 2017;64:S328-s336.

9. Feikin DR, Fu W, Park DE, et al. Is Higher Viral Load in the Upper Respiratory Tract Associated With Severe Pneumonia? Findings From the PERCH Study. *Clin Infect Dis*. 2017;64:S337-s346.

10. Murdoch DR, O'Brien KL, Driscoll AJ, Karron RA, Bhat N. Laboratory methods for determining pneumonia etiology in children. *Clin Infect Dis*. 2012;54 Suppl 2:S146-152.

11. Driscoll AJ, Deloria Knoll M, Hammitt LL, et al. The Effect of Antibiotic Exposure and Specimen Volume on the Detection of Bacterial Pathogens in Children With Pneumonia. *Clin Infect Dis*. 2017;64:S368-s377.

12. Deloria Knoll M, Morpeth SC, Scott JAG, et al. Evaluation of Pneumococcal Load in Blood by Polymerase Chain Reaction for the Diagnosis of Pneumococcal Pneumonia in Young Children in the PERCH Study. *Clin Infect Dis*. 2017;64:S357-s367.

13. Wu Z, Deloria-Knoll M, Zeger SL. Nested partially latent class models for dependent binary data; estimating disease etiology. *Biostatistics*. 2016.

14. Deloria Knoll M, Fu W, Shi Q, et al. Bayesian Estimation of Pneumonia Etiology: Epidemiologic Considerations and Applications to the Pneumonia Etiology Research for Child Health Study. *Clin Infect Dis*. 2017;64:S213-s227.

15. Pneumonia Etiology Research for Child Health (PERCH) Study Group. Causes of severe pneumonia requiring hospital admission in children without HIV infection from Africa and Asia: the PERCH multi-country case-control study. *Lancet*. 2019;394:757-779.

16. Zar HJ, Barnett W, Stadler A, Gardner-Lubbe S, Myer L, Nicol MP. Aetiology of childhood pneumonia in a well vaccinated South African birth cohort: a nested case-control study of the Drakenstein Child Health Study. *Lancet Respir Med*. 2016;4:463-472.

17. Nicol MP, Zar HJ. New specimens and laboratory diagnostics for childhood pulmonary TB: progress and prospects. *Paediatr Respir Rev*. 2011;12:16-21.

18. World Health Organization. Countdown to 2015: Global Tuberculosis Report 2013 Supplement. In: World Health Organization, ed. Geneva: World Health Organization; 2013.
