# Supplementary material for: The Etiology of Pneumonia in HIV-uninfected South African Children: Findings From the Pneumonia Etiology Research for Child Health (PERCH) Study
Source: Pediatr Infect Dis J. 2021 Aug 25;40(9):S59–68. doi: 10.1097/INF.0000000000002650 (PMC8448398; doi:10.1097/INF.0000000000002650)
Supplement: Supplementary file 2 [file inf-40-s59-s002.pdf]

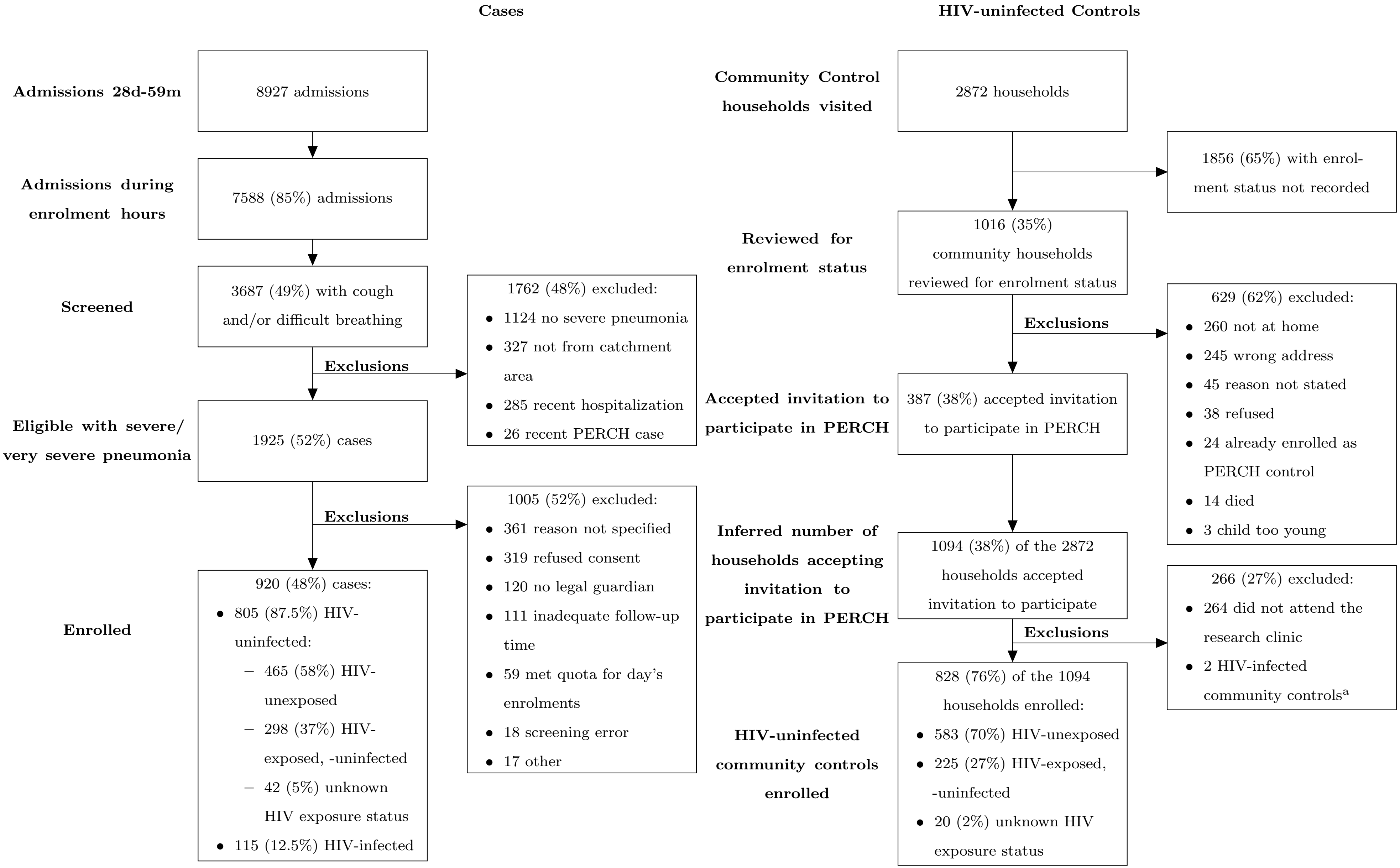

Supplemental Digital Content 2. Case and HIV-uninfected Control Enrolments in PERCH – South African Site

[a] HIV-infected community controls reflect controls enrolled into the PERCH study from the community who were HIV-infected. For analysis purposes, the two HIV-infected community controls were combined with the controls enrolled from HIV clinics to form the full set of HIV-infected controls.
