# Supplementary material for: The Etiology of Pneumonia in HIV-uninfected South African Children: Findings From the Pneumonia Etiology Research for Child Health (PERCH) Study
Source: Pediatr Infect Dis J. 2021 Aug 25;40(9):S59–68. doi: 10.1097/INF.0000000000002650 (PMC8448398; doi:10.1097/INF.0000000000002650)
Supplement: Supplementary file 3 [file inf-40-s59-s003.docx]

## Supplemental Digital Content 3: Demographic and Clinical Characteristics of HIV-exposed, uninfected Cases and Controls Enrolled into PERCH at the South African Site

| Characteristic | All Cases (n=298) | CXR+ Cases (n=165/289) | All Controls (n=225) | OR (95% CI); Adjusted P-value ^a^ | |
| --- | --- | --- | --- | --- | --- |
|  |  |  |  | All Cases compared to Controls | CXR+ Cases compared to Controls |
| Age (months) | | | | | |
| Median age (IQR) | 5.0 (2.0-10.0) | 5.0 (2.0-10.0) | 9.0 (4.0-16.0) | 0.96 (0.94, 0.98); <0.001 | 0.95 (0.93, 0.97); <0.001 |
| Sex | | | | | |
| Female | 141//298 (47.3) | 86/165 (52.1) | 110/225 (48.9) | 0.94 (0.66, 1.34); 0.953 | 1.15 (0.76, 1.75); 0.740 |
| Respiratory Tract Illness (Controls only) ^b^ | | | | | |
|  | - | - | 7/225 (3.1) | - | - |
| Anthropometry | | | | | |
| WAZ ≥-2 | 199/296 (67.2) | 101/163 (62.0) | 206/221 (93.2) | Ref | Ref |
| WAZ ≥-3 to <-2 | 50/296 (16.9) | 31/163 (19.0) | 10/221 (4.5) | 4.96 (2.43, 10.12); <0.001 | 6.15 (2.86, 13.21); <0.001 |
| WAZ <-3 | 47/296 (15.9) | 31/163 (19.0) | 5/221 (2.3) | 8.46 (3.26, 21.96); <0.001 | 11.38 (4.21, 30.76); <0.001 |
| Socio-economic Status | | | | | |
| Lowest tier | 51/298 (17.1) | 31/165 (18.8) | 14/225 (6.2) | 3.16 (1.48, 6.72); 0.010 | 3.54 (1.52, 8.25); 0.009 |
| Low-to-mid tier | 86/296 (28.9) | 49/165 (29.7) | 58/225 (25.8) | 1.54 (0.87, 2.73); 0.282 | 1.63 (0.83, 3.20); 0.277 |
| Mid-to-upper tier | 123/298 (41.3) | 64/165 (38.8) | 115/225 (51.1) | 1.14 (0.67, 1.94); 0.889 | 1.06 (0.57, 2.00); 0.979 |
| Upper tier | 38/298 (12.8) | 21/165 (12.7) | 38/225 (16.9) | Ref | Ref |
| Immunization Status | | | | | |
| BCG Immunization | 267/271 (98.5) | 147/150 (98.0) | 223/223 (100.0) | N/E | N/E |
| DTP-Hib Immunization up-to-date ^c^ | 176/272 (64.7) | 100/151 (66.2) | 153/222 (68.9) | 0.93 (0.63, 1.37); 0.953 | 1.02 (0.65, 1.62); 0.990 |
| PCV Immunization up-to-date ^d^ | 190/272 (69.9) | 109/151 (72.2) | 157/222 (70.7) | 0.81 (0.54, 1.22); 0.534 | 0.90 (0.55, 1.45); 0.840 |
| Measles Immunization up-to-date ^e^ | 70/272 (25.7) | 38/151 (25.2) | 103/222 (46.4) | 0.65 (0.38, 1.11); 0.247 | 0.63 (0.34, 1.18); 0.277 |
| CRP | | | | | |
| Median mg/L (IQR) | 10.8 (2.2-38.9) | 14.1 (3.1-47.0) | 1.0 (0.3-3.5) | 1.13 (1.05, 1.21); 0.003 | 1.14 (1.06, 1.23); <0.001 |
| ≥40 mg/L | 69/292 (23.6) | 47/161 (29.2) | 0/40 (0.0) | N/E | N/E |
| Prior Exposure to Medications | | | | | |
| Serum Antibiotic Activity | 139/279 (49.8) | 85/154 (55.2) | 1/211 (0.5) | 203.19 (28.01, 1.47e+03); <0.001 | 305.65 (39.97, 2.34e+03); <0.001 |

Abbreviations: BCG = Bacillus Calmette-Guérin; CI = Confidence Interval; CRP = C-reactive protein; CXR+ = Radiologically-confirmed pneumonia; DTP = Diphtheria, tetanus, pertussis; Hib = *Haemophilus influenzae* type b; IQR = Interquartile range; N/E = No estimate; OR = Odds ratio; PCV = Pneumococcal conjugate vaccine; PERCH = Pneumonia Etiology Research for Child Health study; Ref = Referent; WAZ = Weight-for-age Z-score.

^a^ Odds ratio adjusted by age (in months) and season, and derived by logistic regression analysis. P-values adjusted using the Benjamini-Hochberg method.

^b^ Respiratory tract illness in PERCH controls was defined as presence of cough or runny nose, or if a child had (1) at least 1 of ear discharge, wheezing, or difficulty breathing and (2) either a measured temperature of >38.0°C within the previous 48 hours or a history of sore throat.

^c^ Complete vaccination defined as receipt of ≥3 doses.

^d^ Complete vaccination defined based on number of doses, and age at first dose, or age at PCV introduction in South Africa: ≥3 doses, or 2 doses if there were at least 8 weeks between doses and the child was <9 months of age at enrolment or >12 months of age at the time of first dose, or ≥1 dose if the age at any of the doses, or age at PCV introduction, was ≥24 months.

^e^ Complete vaccination defined as receipt of at least one dose, restricted to children aged ≥10 months.
