# Supplementary material for: The Etiology of Pneumonia in HIV-uninfected South African Children: Findings From the Pneumonia Etiology Research for Child Health (PERCH) Study
Source: Pediatr Infect Dis J. 2021 Aug 25;40(9):S59–68. doi: 10.1097/INF.0000000000002650 (PMC8448398; doi:10.1097/INF.0000000000002650)
Supplement: Supplementary file 4 [file inf-40-s59-s004.docx]

## Supplemental Digital Content 4: Demographic and Clinical Characteristics of HIV-unexposed Cases and Controls Enrolled into PERCH at the South African Site

| Characteristic | All Cases (n=465) | CXR+ Cases (n=246/443) | All Controls (n=583) | OR (95% CI); Adjusted P-value ^a^ | |
| --- | --- | --- | --- | --- | --- |
|  |  |  |  | All cases compared to Controls | CXR+ Cases compared to Controls |
| Age (months) | | | | | |
| Median age (IQR) | 6.0 (2.0-13.0) | 6.0 (2.0-13.0) | 7.0 (4.0-15.0) | 0.98 (0.96, 0.99); <0.001 | 0.97 (0.96, 0.99); <0.001 |
| Sex | | | | | |
| Female | 218/465 (46.9) | 125/246 (50.8) | 303/583 (52.0) | 0.81 (0.63, 1.03); 0.163 | 0.95 (0.70, 1.28); 0.817 |
| Respiratory Tract Illness (Controls only) ^b^ | | | | | |
|  | - | - | 36/583 (6.2) | - | - |
| Anthropometry | | | | | |
| WAZ ≥-2 | 362/462 (78.4) | 182/243 (74.9) | 559/580 (96.4) | Ref | Ref |
| WAZ ≥-3 to <-2 | 48/462 (10.4) | 31/243 (12.8) | 16/580 (2.8) | 4.78 (2.66, 8.60); <0.001 | 6.18 (3.27, 11.67); <0.001 |
| WAZ <-3 | 52/462 (11.3) | 30/243 (12.3) | 5/580 (0.9) | 15.57 (6.14, 39.47); <0.001 | 17.31 (6.57, 45.62); <0.001 |
| Socio-economic Status | | | | | |
| Lowest tier | 50/465 (10.8) | 31/246 (12.6) | 17/579 (2.9) | 3.48 (1.87, 6.46); <0.001 | 4.69 (2.34, 9.39); <0.001 |
| Low-to-mid tier | 125/465 (26.9) | 73/246 (29.7) | 115/579 (19.9) | 1.28 (0.88, 1.86); 0.335 | 1.61 (1.01, 2.56); 0.086 |
| Mid-to-upper tier | 195/465 (41.9) | 97/246 (39.4) | 332/579 (57.3) | 0.73 (0.53, 1.02); 0.124 | 0.79 (0.52, 1.20); 0.380 |
| Upper tier | 95/465 (20.4) | 45/246 (18.3) | 115/579 (19.9) | Ref | Ref |
| Immunization Status | | | | | |
| BCG Immunization | 435/437 (99.5) | 231/232 (99.6) | 569/572 (99.5) | 1.35 (0.22, 8.20); 0.853 | 1.44 (0.14, 14.32); 0.822 |
| DTP-Hib Immunization up-to-date ^c^ | 286/440 (65.0) | 151/234 (64.5) | 418/572 (73.1) | 0.73 (0.53, 0.96); 0.049 | 0.72 (0.51, 1.00); 0.087 |
| PCV Immunization up-to-date ^d^ | 289/440 (65.7) | 149/234 (63.7) | 431/572 (75.3) | 0.54 (0.40, 0.72); <0.001 | 0.48 (0.34, 0.68); <0.001 |
| Measles Immunization up-to-date ^e^ | 138/438 (31.5) | 72/233 (30.9) | 242/573 (42.2) | 0.94 (0.64, 1.37); 0.853 | 0.90 (0.57, 1.43); 0.817 |
| CRP | | | | | |
| Median mg/L (IQR) | 13.1 (3.0-41.4) | 19.4 (5.6-52.5) | 0.9 (0.3-3.1) | 1.17 (1.11, 1.23); <0.001 | 1.17 (1.11, 1.23); <0.001 |
| ≥40 mg/L | 117/452 (25.9) | 79/239 (33.1) | 0/105 (0.0) | N/E | N/E |
| Prior Exposure to Medications | | | | | |
| Serum Antibiotic Activity | 217/448 (48.4) | 127/236 (53.8) | 6/534 (1.1) | 83.17 (36.32, 190.44); <0.001 | 99.28 (42.55, 231.63); <0.001 |

Abbreviations: BCG = Bacillus Calmette-Guérin; CI = Confidence Interval; CRP = C-reactive protein; CXR+ = Radiologically-confirmed pneumonia; DTP = Diphtheria, tetanus, pertussis; Hib = *Haemophilus influenzae* type b; IQR = Interquartile range; N/E = No estimate; OR = Odds ratio; PCV = Pneumococcal conjugate vaccine; PERCH = Pneumonia Etiology Research for Child Health study; Ref = Referent; WAZ = Weight-for-age Z-score.

^a^ Odds ratio adjusted by age (in months) and season, and derived by logistic regression analysis. P-values adjusted using the Benjamini-Hochberg method.

^b^ Respiratory tract illness in PERCH controls was defined as presence of cough or runny nose, or if a child had (1) at least 1 of ear discharge, wheezing, or difficulty breathing and (2) either a measured temperature of >38.0°C within the previous 48 hours or a history of sore throat.

^c^ Complete vaccination defined as receipt of ≥3 doses.

^d^ Complete vaccination defined based on number of doses, and age at first dose, or age at PCV introduction in South Africa: ≥3 doses, or 2 doses if there were at least 8 weeks between doses and the child was <9 months of age at enrolment or >12 months of age at the time of first dose, or ≥1 dose if the age at any of the doses, or age at PCV introduction, was ≥24 months.

^e^ Complete vaccination defined as receipt of at least one dose, restricted to children aged ≥10 months.
