# Supplementary material for: The Etiology of Pneumonia in HIV-uninfected South African Children: Findings From the Pneumonia Etiology Research for Child Health (PERCH) Study
Source: Pediatr Infect Dis J. 2021 Aug 25;40(9):S59–68. doi: 10.1097/INF.0000000000002650 (PMC8448398; doi:10.1097/INF.0000000000002650)
Supplement: Supplementary file 5 [file inf-40-s59-s005.docx]

## Supplemental Digital Content 5: Clinical Characteristics of HIV-uninfected Cases, Stratified by Radiologic Findings and HIV-exposure Status

|  | HIV-uninfected Children | | | HIV-exposed Children | | | HIV-unexposed Children | | |
| --- | --- | --- | --- | --- | --- | --- | --- | --- | --- |
| Characteristic | All Cases (n=805) | Cases with normal CXR (n=246) | CXR+ Cases (n=435) | All Cases (n=298) | Cases with normal CXR (n=94) | CXR+ Cases  (n=165) | All Cases (n=465) | Cases with normal CXR (n=140) | CXR+ Cases  (n=246) |
| Very severe pneumonia ^a^ | 259/805 (32.2) | 67/246  (27.2) | 153/435 (35.2) | 100/298 (33.6) | 24/94  (25.5) | 64/165  (38.8) | 145/465 (31.2) | 39/140  (27.9) | 80/246  (32.5) |
| CXR available | 771/805 (95.8) | 246/246 (100.0) | 435/435 (100.0) | 289/298 (97.0) | 94/94  (100.0) | 165/165 (100.0) | 443/465 (95.3) | 140/140 (100.0) | 246/246 (100.0) |
| CXR Result | | | | | | | | | |
| Any consolidation | 250/771 (32.4) | 0/246  (0.0) | 250/435 (57.5) | 89/289  (30.8) | 0/94  (0.0) | 89/165  (53.9) | 150/443 (33.9) | 0/140  (0.0) | 150/246 (61.0) |
| Other infiltrate only | 185/771 (24.0) | 0/246  (0.0) | 185/435 (42.5) | 76/289  (26.3) | 0/94  (0.0) | 76/165  (46.1) | 96/443 (21.7) | 0/140  (0.0) | 96/246  (39.0) |
| Normal | 246/771 (31.9) | 246/246 (100.0) | 0/435  (0.0) | 94/289  (32.5) | 94/94  (100.0) | 0/165  (0.0) | 140/443 (31.6) | 140/140 (100.0) | 0/246  (0.0) |
| Uninterpretable | 90/771 (11.7) | 0/246 (0.0) | 0/435 (0.0) | 30/289 (10.4) | 0/94 (0.0) | 0/165 (0.0) | 57/443 (12.9) | 0/140 (0.0) | 0/246 (0.0) |
| Laboratory Results | | | | | | | | | |
| Severe anemia ^b^ | 12/803 (1.5) | 2/246 (0.8) | 8/434 (1.8) | 3/298 (1.0) | 1/94 (1.1) | 2/165 (1.2) | 9/463 (1.9) | 1/140 (0.7) | 6/245 (2.4) |
| Leukocytosis ^c^ | 355/803 (44.2) | 113/245 (46.1) | 194/434 (44.7) | 132/298 (44.3) | 48/94  (51.1) | 67/165  (40.6) | 201/463 (43.4) | 58/139  (41.7) | 114/245 (46.5) |
| Leukopenia ^d^ | 8/803 (1.0) | 1/245 (0.4) | 6/434 (1.4) | 3/298 (1.0) | 0/94 (0.0) | 2/165 (1.2) | 4/463 (0.9) | 1/139 (0.7) | 3/245 (1.2) |
| Clinical Parameters | | | | | | | | | |
| Severely Underweight ^e^ | 109/805 (13.5) | 20/246  (8.1) | 71/435  (16.3) | 47/298  (15.8) | 10/94  (10.6) | 31/165  (18.8) | 52/465  (11.2) | 10/140  (7.1) | 30/246  (12.2) |
| Hypoxia ^f^ | 601/801 (75.0) | 179/243 (73.7) | 329/434 (75.8) | 214/298 (71.8) | 64/94  (68.1) | 124/165 (75.2) | 355/461 (77.0) | 104/137 (75.9) | 188/245 (76.7) |
| Fever ≥38°C | 505/805 (62.7) | 124/246 (50.4) | 298/435 (68.5) | 184/298 (61.7) | 44/94  (46.8) | 110/165 (66.7) | 290/465 (62.4) | 69/140  (49.3) | 172/246 (69.9) |
| Tachycardia | 393/804 (48.9) | 122/246 (49.6) | 210/435 (48.3) | 143/298 (48.0) | 42/94  (44.7) | 82/165  (49.7) | 229/464 (49.4) | 72/140  (51.4) | 119/246 (48.4) |
| Wheeze on auscultation | 271/786 (34.5) | 104/244 (42.6) | 122/421 (29.0) | 82/292  (28.1) | 35/94  (37.2) | 35/159  (22.0) | 173/452 (38.3) | 65/138  (47.1) | 80/238  (33.6) |
| Lethargy ^g^ | 41/805 (5.1) | 13/246 (5.3) | 21/435 (4.8) | 20/298 (6.7) | 8/94 (8.5) | 9/165 (5.5) | 20/465 (4.3) | 5/140 (3.6) | 11/246 (4.5) |
| Duration of illness ^h^ | | | | | | | | | |
| Median duration of illness (days; IQR) | 3.0 (2.0, 4.0) | 3.0 (2.0, 4.0) | 3.0 (2.0, 5.0) | 3.0 (2.0, 4.0) | 3.0 (2.0, 4.0) | 3.0 (2.0, 4.0) | 3.0 (2.0, 4.0) | 3.0 (2.0, 4.0) | 3.0 (2.0, 5.0) |
| 0-2 days | 330/787 (41.9) | 115/240  (47.9) | 162/426 (38.0) | 23/298  (7.7) | 45/91  (49.5) | 7/165  (4.2) | 193/454 (42.5) | 64/138  (46.4) | 93/240  (38.8) |
| 3-5 days | 325/787 (41.3) | 92/240  (38.3) | 191/426 (44.8) | 97/298  (32.6) | 32/91  (35.2) | 45/165  (27.3) | 187/454 (41.2) | 56/138  (40.6) | 106/240 (44.2) |
| >5 days | 132/787 (16.8) | 33/240  (13.8) | 73/426  (17.1) | 52/292  (17.8) | 14/91  (15.4) | 28/162  (17.3) | 74/454  (16.3) | 18/138  (13.0) | 41/240  (17.1) |
| Duration of hospitalization | | | | | | | | | |
| Median duration of hospitalization (days; IQR) | 6.0  (4.0, 9.0) | 5.0  (3.0, 7.0) | 7.0  (4.0, 11.0) | 7.0  (4.0, 11.0) | 5.0  (4.0, 9.0) | 7.0  (5.0, 13.0) | 5.5  (4.0, 8.0) | 4.5  (3.0, 7.0) | 7.0  (4.0, 10.0) |
| 0-2 days | 76/804 (9.5) | 26/246 (10.6) | 30/434 (6.9) | 23/298 (7.7) | 8/94 (8.5) | 7/165 (4.2) | 49/464 (10.6) | 17/140 (12.1) | 20/245 (8.2) |
| 3-5 days | 293/804 (36.4) | 122/246 (49.6) | 126/434 (29.0) | 97/298  (32.6) | 42/94  (44.7) | 45/165  (27.3) | 183/464 (39.4) | 74/140  (52.9) | 75/245  (30.6) |
| >5 days | 435/804 (54.1) | 98/246  (39.8) | 278/434 (64.1) | 178/298 (59.7) | 44/94  (46.8) | 113/165 (68.5) | 232/464 (50.0) | 49/140  (35.0) | 150/245 (61.2) |
| Died in Hospital | 20/804 (2.5) | 0/246 (0.0) | 16/434 (3.7) | 8/298 (2.7) | 0/94 (0.0) | 6/165 (3.6) | 11/464 (2.4) | 0/140 (0.0) | 9/245 (3.7) |
| Died post-discharge, within 30 days of admission ^i^ | 5/726  (0.7) | 0/246  (0.0) | 2/391  (0.5) | 3/272  (1.1) | 0/83  (0.0) | 0/154  (0.0) | 1/416  (0.2) | 0/127  (0.0) | 1/215  (0.5) |
| Missing 30-day vital status ^j^ | 119/784 (15.2) | 32/246  (13.0) | 75/418  (17.9) | 48/290  (16.6) | 15/94  (16.0) | 28/159  (17.6) | 62/453  (13.7) | 14/140  (10.0) | 41/236  (17.4) |

Abbreviations: CXR = Chest radiograph; CXR+ = Radiologically-confirmed pneumonia; HIV = Human immunodeficiency virus type-1; IQR = Interquartile range.

^a^ Very severe pneumonia defined as cough or difficulty breathing, and at least one of the following: central cyanosis, difficulty breastfeeding/drinking, vomiting everything, convulsions, lethargy, unconsciousness, or head nodding.

^b^ Severe anemia defined as hemoglobin <7.5 g/dL.

^c^ Defined as leukocyte count >15 × 10^9^ cells/L for children 1-11 months, and >13 × 10^9^ cells/L for children 12-59 months.

^d^ Defined as leukocyte count <5 × 10^9^ cells/L, regardless of age category.

^e^ Severely underweight defined as weight-for-age Z-score <-3, as per World Health Organization Growth Standards.

^f^ Hypoxemia defined as room air oxygen saturation <90%, or on supplemental oxygen if a room air oxygen saturation reading was not available.

^g^ Lethargic or unresponsive (responds to voice or pain, unresponsive, or pharmacologically sedated).

^h^ Duration of illness defined as duration (in days) of cough, wheeze, fever, or difficulty breathing, whichever is longest.

^i^ Restricted to those children with known vital status at 30 days post-admission.

^j^ Restricted to those children discharged alive.
