# Supplementary material for: The Etiology of Pneumonia in HIV-uninfected South African Children: Findings From the Pneumonia Etiology Research for Child Health (PERCH) Study
Source: Pediatr Infect Dis J. 2021 Aug 25;40(9):S59–68. doi: 10.1097/INF.0000000000002650 (PMC8448398; doi:10.1097/INF.0000000000002650)
Supplement: Supplementary file 6 [file inf-40-s59-s006.docx]

## Supplemental Digital Content 6: Microbiology and Mantoux Results in HIV-uninfected Cases

|  | All cases (n=805) | HIV-exposed, uninfected Cases (n=298) | HIV-unexposed Cases (n=465) |
| --- | --- | --- | --- |
| Blood Cultures | | | |
| Total blood cultures | 802/805 (99.6) | 296/298 (99.3) | 464/465 (99.8) |
| Contaminants | 106/802 (13.2) | 36/296 (12.2) | 59/464 (12.7) |
| Significant isolates | 17/802 (2.1) | 5/296 (1.7) | 12/464 (2.6) |
| Gram negatives | 10/17 (58.8) | 2/5 (40.0) | 8/12 (66.7) |
| *E. coli* | 3 | 0 | 3 |
| *H. influenzae* | 2 | 1 | 1 |
| *K. pneumoniae* | 1 | 0 | 1 |
| *M. catarrhalis* | 1 | 0 | 1 |
| *N. meningitidis* | 1 | 0 | 1 |
| *P. aeruginosa* | 1 | 1 | 0 |
| Salmonella spp. | 1 | 0 | 1 |
| Gram positives | 7/17 (41.2) | 3/5 (60.0) | 4/12 (33.3) |
| *E. faecium* | 2 | 2 | 0 |
| *S. aureus* | 5 | 1 | 4 |
| Flag positive, blood culture negative | | | |
| Pneumococcal latex positive | 1 | 0 | 1 |
| Pleural Fluid Cultures | | | |
| No organism detected | 4 | 2 | 2 |
| *S. aureus* | 1 | 1 | 0 |
| Pleural Fluid PCR | | | |
| No organism detected | 2 | 1 | 1 |
| *S. pneumoniae* | 2 | 1 | 1 |
| *S. aureus* | 1 | 1 | 0 |
| Lung Aspirates | | | |
| Lung aspirate PJP microscopy | 1/5 (20.0) | 0/2 (0.0) | 1/3 (33.3) |
| Pos PJP Immunofluorescence | 0/1 (0.0) | - | 0/1 (0.0) |
| Lung aspirate cultures | | | |
| No organism detected | 5 | 2 | 3 |
| Lung aspirate PCR | | | |
| No organism detected | 2 | 1 | 1 |
| ADENO, CPNEU | 1 | 1 | 0 |
| HMPV, HINF, MCAT | 1 | 0 | 1 |
| HINF, PNEU | 1 | 0 | 1 |
| Induced Sputum Specimens (IS) | | | |
| Number of children with IS Specimens | 740/805 (91.9) | 269/298 (90.3) | 433/465 (93.1) |
| IS PJP microscopy | 550/740 (74.3) | 190/269 (70.6) | 329/433 (76.0) |
| Pos PJP Immunofluorescence | 2/550 (0.4) | 1/190 (0.5) | 1/329 (0.3) |
| IS *Mtb* Culture Pos | 13/740 (1.8) | 8/269 (3.0) | 5/433 (1.2) |
| Gastric Aspirate Samples (GA) | | | |
| Number of children with GA Specimens | 419/805 (52.0) | 154/298 (51.7) | 243/465 (52.3) |
| GA *Mtb* Culture Pos | 14/418 (3.3) | 6/154 (3.9) | 8/243 (3.3) |
| Endotracheal Tube Specimens (ETT) | | | |
| Number of children with ETT Specimens | 65/805 (8.1) | 30/298 (10.1) | 32/465 (6.9) |
| ETT PJP microscopy | 37/65 (56.9) | 18/30 (60.0) | 18/32 (56.2) |
| Pos PJP Immunofluorescence | 0/37 (0.0) | 0/18 (0.0) | 0/18 (0.0) |
| ETT *Mtb* Culture Pos | 1/65 (1.5) | 1/30 (3.3) | 0/32 (0.0) |
| Tuberculin Skin Tests | | | |
| Total Mantoux tests | 414/805 (51.4) | 147/298 (49.3) | 243/465 (52.3) |
| Neg | 363/414 (87.7) | 127/147 (86.4) | 213/243 (87.7) |
| Pos | 51/414 (12.3) | 20/147 (13.6) | 30/243 (12.3) |
| Certainty of tuberculosis diagnosis | | | |
| Clinical tuberculosis* | 74/804 (9.2) | 26/298 (8.7) | 45/464 (9.7) |
| Definite tuberculosis‡ | 24/805 (2.9) | 12/298 (4.0) | 12/465 (2.6) |

Abbreviations: ADENO = Adenovirus; CPNEU = *Chlamydophila pneumoniae*; ETT = Endotracheal tube; HEU = HIV-exposed, -uninfected; HINF = *Haemophilus influenzae* non-type b; HIV = Human immunodeficiency virus type-1; HMPV = Human metapneumovirus; GA = Gastric aspirate; IS = Induced sputum; MCAT = *Moraxella catarrhalis*; *Mtb* = *Mycobacterium tuberculosis*; Neg = Negative; PJP = *Pneumocystis jirovecii*; PNEU = *Streptococcus pneumoniae*; Pos = Positive.

* Clinical tuberculosis: Tuberculosis diagnosed clinically, based either on the presence of a suggestive chest X-ray, and/or positive Mantoux reaction, and/or family history of exposure to a case of tuberculosis.

‡ Definite tuberculosis: *Mtb* cultured on respiratory specimens or mycobacterial blood culture. Twenty-three children had *Mtb* cultured on GA and/or IS specimens (respiratory specimens), and one (HIV-exposed, uninfected) had *Mtb* cultured on blood culture only.

Results are not restricted to children with radiologically-confirmed pneumonia.
