# Supplementary material for: The Etiology of Pneumonia in HIV-uninfected South African Children: Findings From the Pneumonia Etiology Research for Child Health (PERCH) Study
Source: Pediatr Infect Dis J. 2021 Aug 25;40(9):S59–68. doi: 10.1097/INF.0000000000002650 (PMC8448398; doi:10.1097/INF.0000000000002650)
Supplement: Supplementary file 7 [file inf-40-s59-s007.docx]

## Supplemental Digital Content 7: Conditional Odds Ratios in the Comparison between All Cases, Cases with Radiologically-confirmed Pneumonia, and Controls: HIV-uninfected Children

| Pathogen | All Cases | CXR+ Cases | Controls | Conditional Odds Ratio (95% CI) ^a^ | |
| --- | --- | --- | --- | --- | --- |
|  |  |  |  | All Cases vs. Controls | CXR+ Cases vs. Controls |
| Any non-viral pathogen | 742/802 (92.5) | 406/433 (93.8) | 774/823 (94.0) | 0.80 (0.54, 1.19) | 0.97 (0.59, 1.59) |
| Any non-viral pathogen, above cut-off density threshold ^b^ | 647/802 (80.7) | 353/433 (81.5) | 677/823 (82.3) | 0.88 (0.68, 1.14) | 0.93 (0.68, 1.26) |
| Bacteria | | | | | |
| *Bordetella pertussis* | 17/802 (2.1) | 10/433 (2.3) | 4/823 (0.5) | **4.47 (1.43**, **13.99)** | **6.15 (1.79**, **21.04)** |
| *Chlamydophila pneumoniae* | 14/802 (1.7) | 9/433 (2.1) | 20/823 (2.4) | 0.85 (0.38, 1.89) | 0.85 (0.32, 2.28) |
| *Haemophilus influenzae* type b | 12/802 (1.5) | 6/433 (1.4) | 7/823 (0.9) | 2.15 (0.78, 5.87) | 2.22 (0.68, 7.25) |
| *Haemophilus influenzae* type b ≥ threshold density ^c^ | 5/802 (0.6) | 3/433 (0.7) | 2/823 (0.2) | 1.37 (0.96, 1.97) | **1.55 (1.01**, **2.39)** |
| Non-type b *Haemophilus influenzae* | 413/802 (51.5) | 255/433 (58.9) | 389/823 (47.3) | 1.18 (0.93, 1.50) | **1.61 (1.20**, **2.16)** |
| Non-type b *Haemophilus influenzae* ≥ threshold density ^c^ | 224/802 (27.9) | 145/433 (33.5) | 182/823 (22.1) | **1.30 (1.00**, **1.70)** | **1.62 (1.18**, **2.22)** |
| *Moraxella catarrhalis* | 481/802 (60.0) | 269/433 (62.1) | 548/823 (66.6) | 0.81 (0.64, 1.03) | 0.91 (0.67, 1.22) |
| *Mycoplasma pneumoniae* | 6/794 (0.8) | 2/428 (0.5) | 5/823 (0.6) | 1.43 (0.35, 5.81) | 0.96 (0.11, 7.94) |
| *Streptococcus pneumoniae* | 511/802 (63.7) | 290/433 (67.0) | 568/823 (69.0) | 0.87 (0.68, 1.12) | 0.91 (0.66, 1.24) |
| *Streptococcus pneumoniae* ≥ threshold density ^d^ | 87/802 (10.8) | 55/433 (12.7) | 80/823 (9.7) | 0.89 (0.60, 1.30) | 0.98 (0.62, 1.54) |
| Vaccine type *Streptococcus pneumoniae* ^e^ | 30/802 (3.7) | 20/433 (4.6) | 30/826 (3.6) | 0.86 (0.46, 1.59) | 1.16 (0.58, 2.33) |
| Non-vaccine type *Streptococcus pneumoniae* ^e^ | 57/802 (7.1) | 37/433 (8.5) | 53/827 (6.4) | 0.88 (0.56, 1.40) | 0.94 (0.55, 1.61) |
| *Streptococcus pneumoniae* in whole blood | 57/802 (7.1) | 36/432 (8.3) | 87/827 (10.5) | 0.77 (0.52, 1.14) | 0.91 (0.57, 1.47) |
| *Streptococcus pneumoniae* in whole blood ≥ threshold density ^f^ | 42/802 (5.2) | 27/432 (6.2) | 42/827 (5.1) | 1.33 (0.81, 2.20) | 1.63 (0.91, 2.95) |
| Salmonella spp | 0/802 (0.0) | 0/433 (0.0) | 0/823 (0.0) | N/E | N/E |
| *Staphylococcus aureus* | 206/802 (25.7) | 98/433 (22.6) | 149/823 (18.1) | **1.39 (1.05**, **1.83)** | 1.15 (0.81, 1.63) |
| Fungal species | | | | | |
| *Pneumocystis jirovecii* | 100/802 (12.5) | 63/433 (14.5) | 88/823 (10.7) | 1.10 (0.78, 1.56) | 1.43 (0.95, 2.18) |
| *Pneumocystis jirovecii* ≥ threshold density ^g^ | 55/802 (6.9) | 31/433 (7.2) | 25/823 (3.0) | **2.55 (1.50**, **4.32)** | **2.67 (1.45**, **4.93)** |
| Viruses | | | | | |
| Any viral pathogen | 677/802 (84.4) | 380/433 (87.8) | 613/823 (74.5) | **1.85 (1.44**, **2.38)** | **2.42 (1.74**, **3.38)** |
| Any viral pathogen, above cut-off density threshold ^b^ | 651/802 (81.2) | 370/433 (85.5) | 561/823 (68.2) | **1.97 (1.56**, **2.48)** | **2.64 (1.94**, **3.60)** |
| Adenovirus | 79/795 (9.9) | 47/429 (11.0) | 84/823 (10.2) | 1.37 (0.96, 1.97) | **1.55 (1.01**, **2.39)** |
| Human cytomegalovirus | 291/795 (36.6) | 167/429 (38.9) | 378/823 (45.9) | 0.76 (0.61, 0.96) | 0.87 (0.66, 1.16) |
| Human cytomegalovirus ≥ threshold density ^h^ | 160/795 (20.1) | 101/429 (23.5) | 212/823 (25.8) | 0.65 (0.49, 0.85) | 0.85 (0.61, 1.18) |
| Coronavirus 229 | 3/795 (0.4) | 1/429 (0.2) | 2/823 (0.2) | 1.79 (0.25, 13.05) | 1.31 (0.10, 16.84) |
| Coronavirus 43 | 25/795 (3.1) | 12/429 (2.8) | 46/823 (5.6) | 0.50 (0.28, 0.89) | 0.55 (0.26, 1.15) |
| Coronavirus 63 | 22/795 (2.8) | 11/429 (2.6) | 26/823 (3.2) | 1.46 (0.78, 2.73) | 1.46 (0.67, 3.18) |
| Coronavirus HKU | 11/795 (1.4) | 9/429 (2.1) | 18/823 (2.2) | 0.81 (0.35, 1.88) | 1.34 (0.52, 3.43) |
| Influenza A | 28/795 (3.5) | 17/429 (4.0) | 13/823 (1.6) | **4.29 (2.09**, **8.80)** | **4.80 (2.13**, **10.83)** |
| Influenza B | 9/795 (1.1) | 7/429 (1.6) | 3/823 (0.4) | **4.62 (1.13**, **18.95)** | **8.10 (1.80**, **36.42)** |
| Influenza C | 7/802 (0.9) | 5/433 (1.2) | 6/823 (0.7) | 1.51 (0.44, 5.14) | 1.87 (0.43, 8.20) |
| Human bocavirus | 84/794 (10.6) | 45/428 (10.5) | 83/823 (10.1) | 1.22 (0.84, 1.77) | 1.32 (0.82, 2.11) |
| Human metapneumovirus A/B | 52/794 (6.5) | 31/428 (7.2) | 27/823 (3.3) | **2.76 (1.65**, **4.62)** | **3.39 (1.88**, **6.13)** |
| Parainfluenza virus 1 | 16/794 (2.0) | 9/428 (2.1) | 2/823 (0.2) | **14.09 (3.17**, **62.58)** | **17.42 (3.61**, **83.96)** |
| Parainfluenza virus 2 | 1/795 (0.1) | 1/429 (0.2) | 8/823 (1.0) | 0.18 (0.02, 1.49) | 0.38 (0.05, 3.17) |
| Parainfluenza virus 3 | 42/795 (5.3) | 26/429 (6.1) | 17/823 (2.1) | **3.84 (2.11**, **7.00)** | **4.56 (2.35**, **8.88)** |
| Parainfluenza virus 4 | 16/795 (2.0) | 9/429 (2.1) | 12/823 (1.5) | 1.53 (0.66, 3.56) | 1.86 (0.68, 5.03) |
| Parechovirus/Enterovirus | 46/795 (5.8) | 23/429 (5.4) | 62/823 (7.5) | 1.06 (0.68, 1.66) | 0.86 (0.48, 1.53) |
| Human rhinovirus | 178/795 (22.4) | 84/429 (19.6) | 187/823 (22.7) | **1.44 (1.10**, **1.89)** | 1.15 (0.82, 1.62) |
| Respiratory syncytial virus | 229/795 (28.8) | 141/429 (32.9) | 27/823 (3.3) | **14.36 (9.34**, **22.07)** | **18.14 (11.48**, **28.66)** |

Abbreviations: CI = Confidence Interval; CXR+ = Radiologically-confirmed pneumonia; HIV = Human immunodeficiency virus type-1; N/E = No estimate; NP/OP = Nasopharyngeal/oropharyngeal.

^a^ Conditional odds ratio derived by logistic regression, adjusting age (in months) and presence of all other pathogens: two analyses were combined in the output of this Table: the first with no threshold applied for human cytomegalovirus, *H. influenzae*, *P. jirovecii*, and *S. pneumoniae*, and the second with threshold density cut-offs (as noted below) applied to these pathogens. The first analysis output was used to report the adjusted conditional odds for cytomegalovirus, *H. influenzae*, *P. jirovecii*, and *S. pneumoniae* with no threshold density cut-off applied. The second analysis output was used to report the adjusted conditional odds for all pathogens named in the Table.

^b^ Cut-off density threshold which best distinguished between cases and controls, derived by receiver operating characteristic analysis using leave-one-out cross-validation.

^c^ Cut-off density for *H. influenzae* (non-type b, and type b) on NP/OP swabs: 5.9 log_10_ copies/mL.

^d^ Cut-off density for *S. pneumoniae* on NP/OP swabs: 6.9 log_10_ copies/mL.

^e^ Vaccine-type pneumococcus amongst children with high density NP/OP pneumococcal carriage.

^f^ Cut-off density for *S. pneumoniae* in whole blood specimens: 2.2 log_10_ copies/mL.

^g^ Cut-off density for *P. jirovecii* on NP/OP swabs: 4.0 log_10_ copies/mL.

^h^ Cut-off density for human cytomegalovirus on NP/OP swabs: 4.9 log_10_ copies/mL.
