# Supplementary material for: The Etiology of Pneumonia in HIV-uninfected South African Children: Findings From the Pneumonia Etiology Research for Child Health (PERCH) Study
Source: Pediatr Infect Dis J. 2021 Aug 25;40(9):S59–68. doi: 10.1097/INF.0000000000002650 (PMC8448398; doi:10.1097/INF.0000000000002650)
Supplement: Supplementary file 8 [file inf-40-s59-s008.docx]

***Supplemental Digital Content 8: Conditional Odds Ratios in the Comparison between Cases Dying In-hospital, and Controls: HIV-exposed, -uninfected Children***

| Pathogen | Cases Dying In-hospital | | Controls | Conditional Odds Ratio (95% CI) ^a^ |
| --- | --- | --- | --- | --- |
|  |  |  |  | Cases Dying In-hospital vs. Controls |
| Any non-viral pathogen | 7/8 (87.5) | | 210/224 (93.8) | 0.49 (0.05, 4.37) |
| Any non-viral pathogen, above cut-off density threshold ^b^ | 6/8 (75.0) | | 191/224 (85.3) | 0.46 (0.09, 2.45) |
| Bacteria | | | | |
| *Bordetella pertussis* | 0/8 (0.0) | | 1/224 (0.4) | N/E |
| *Chlamydophila pneumoniae* | 0/8 (0.0) | | 3/224 (1.3) | N/E |
| *Haemophilus influenzae* type b | 0/8 (0.0) | | 2/224 (0.9) | N/E |
| *Haemophilus influenzae* type b ≥ threshold density ^c^ | 0/8 (0.0) | | 0/224 (0.0) | N/E |
| Non-type b *Haemophilus influenzae* | 2/8 (25.0) | | 111/224 (49.6) | 0.46 (0.05, 4.23) |
| Non-type b *Haemophilus influenzae* ≥ threshold density ^c^ | 1/8 (12.5) | | 57/224 (25.4) | 0.62 (0.05, 7.77) |
| *Moraxella catarrhalis* | 2/8 (25.0) | | 149/224 (66.5) | 0.27 (0.03, 2.49) |
| *Mycoplasma pneumoniae* | 0/8 (0.0) | | 2/224 (0.9) | N/E |
| *Streptococcus pneumoniae* | 4/8 (50.0) | | 155/224 (69.2) | 0.45 (0.05, 4.10) |
| *Streptococcus pneumoniae* ≥ threshold density ^d^ | 1/8 (12.5) | | 23/224 (10.3) | 2.59 (0.08, 88.13) |
| Vaccine type *Streptococcus pneumoniae* ^e^ | 0/8 (0.0) | | 8/224 (3.6) | N/E |
| Non-vaccine type *Streptococcus pneumoniae* ^e^ | 1/8 (12.5) | | 15/225 (6.7) | 4.26 (0.10, 182.43) |
| *Streptococcus pneumoniae* in whole blood | 0/8 (0.0) | | 23/225 (10.2) | N/E |
| *Streptococcus pneumoniae* in whole blood ≥ threshold density ^f^ | 0/8 (0.0) | | 11/225 (4.9) | N/E |
| Salmonella spp | 0/8 (0.0) | | 0/224 (0.0) | N/E |
| *Staphylococcus aureus* | 3/8 (37.5) | | 40/224 (17.9) | 0.91 (0.06, 13.74) |
| Fungal species | | | | |
| *Pneumocystis jirovecii* | 3/8 (37.5) | | 21/224 (9.4) | 4.78 (0.49, 46.24) |
| *Pneumocystis jirovecii* ≥ threshold density ^g^ | 2/8 (25.0) | | 5/224 (2.2) | **70.19 (2.58**, **1910.37)** |
| Viruses | | | | |
| Any viral pathogen | | 6/8 (75.0) | 158/224 (70.5) | 1.30 (0.25, 6.74) |
| Any viral pathogen, above cut-off density threshold ^b^ | | 6/8 (75.0) | 141/224 (62.9) | 1.93 (0.37, 10.12) |
| Adenovirus | | 0/8 (0.0) | 38/224 (17.0) | N/E |
| Human cytomegalovirus | | 5/8 (62.5) | 74/224 (33.0) | 3.39 (0.57, 20.10) |
| Human cytomegalovirus ≥ threshold density ^h^ | | 4/8 (50.0) | 32/224 (14.3) | 8.58 (0.65, 114.07) |
| Coronavirus 229 | | 0/8 (0.0) | 0/224 (0.0) | N/E |
| Coronavirus 43 | | 0/8 (0.0) | 17/224 (7.6) | N/E |
| Coronavirus 63 | | 1/8 (12.5) | 3/224 (1.3) | 20.50 (0.61, 683.52) |
| Coronavirus HKU | | 0/8 (0.0) | 3/224 (1.3) | N/E |
| Influenza A | | 0/8 (0.0) | 3/224 (1.3) | N/E |
| Influenza B | | 0/8 (0.0) | 1/224 (0.4) | N/E |
| Influenza C | | 0/8 (0.0) | 0/224 (0.0) | N/E |
| Human bocavirus | | 1/8 (12.5) | 21/224 (9.4) | 3.55 (0.14, 93.00) |
| Human metapneumovirus A/B | | 2/8 (25.0) | 7/224 (3.1) | **18.61 (1.00**, **345.74)** |
| Parainfluenza virus 1 | | 0/8 (0.0) | 0/224 (0.0) | N/E |
| Parainfluenza virus 2 | | 0/8 (0.0) | 2/224 (0.9) | N/E |
| Parainfluenza virus 3 | | 0/8 (0.0) | 5/224 (2.2) | N/E |
| Parainfluenza virus 4 | | 0/8 (0.0) | 2/224 (0.9) | N/E |
| Parechovirus/Enterovirus | | 1/8 (12.5) | 19/224 (8.5) | 2.66 (0.13, 52.94) |
| Human rhinovirus | | 0/8 (0.0) | 50/224 (22.3) | N/E |
| Respiratory syncytial virus | | 1/8 (12.5) | 6/224 (2.7) | 5.28 (0.15, 187.57) |

Abbreviations: CI = Confidence Interval; CXR+ = Radiologically-confirmed pneumonia; N/E = No estimate; NP/OP = Nasopharyngeal/oropharyngeal.

^a^ Conditional odds ratio derived by logistic regression, adjusting age (in months) and presence of all other pathogens: two analyses were combined in the output of this Table: the first with no threshold applied for human cytomegalovirus, *H. influenzae*, *P. jirovecii*, and *S. pneumoniae*, and the second with threshold density cut-offs (as noted below) applied to these pathogens. The first analysis output was used to report the adjusted conditional odds for cytomegalovirus, *H. influenzae*, *P. jirovecii*, and *S. pneumoniae* with no threshold density cut-off applied. The second analysis output was used to report the adjusted conditional odds for all pathogens named in the Table.

^b^ Cut-off density threshold which best distinguished between cases and controls, derived by receiver operating characteristic analysis using leave-one-out cross-validation.

^c^ Cut-off density for *H. influenzae* (non-type b, and type b) on NP/OP swabs: 5.9 log_10_ copies/mL.

^d^ Cut-off density for *S. pneumoniae* on NP/OP swabs: 6.9 log_10_ copies/mL.

^e^ Vaccine-type pneumococcus amongst children with high density NP/OP pneumococcal carriage.

^f^ Cut-off density for *S. pneumoniae* in whole blood specimens: 2.2 log_10_ copies/mL.

^g^ Cut-off density for *P. jirovecii* on NP/OP swabs: 4.0 log_10_ copies/mL.

^h^ Cut-off density for human cytomegalovirus on NP/OP swabs: 4.9 log_10_ copies/mL.
