# Supplementary material for: The Etiology of Pneumonia in HIV-uninfected South African Children: Findings From the Pneumonia Etiology Research for Child Health (PERCH) Study
Source: Pediatr Infect Dis J. 2021 Aug 25;40(9):S59–68. doi: 10.1097/INF.0000000000002650 (PMC8448398; doi:10.1097/INF.0000000000002650)
Supplement: Supplementary file 9 [file inf-40-s59-s009.docx]

***Supplemental Digital Content 9: Conditional Odds Ratios in the Comparison between Cases Dying In-hospital, and Controls: HIV-unexposed Children***

| Pathogen | Cases Dying In-hospital | | Controls | Conditional Odds Ratio (95% CI) ^a^ |
| --- | --- | --- | --- | --- |
|  |  |  |  | Cases Dying In-hospital vs. Controls |
| Any non-viral pathogen | 11/11 (100.0) | | 545/579 (94.1) | N/E |
| Any non-viral pathogen, above cut-off density threshold ^b^ | 10/11 (90.9) | | 469/579 (81.0) | 2.46 (0.31, 19.55) |
| Bacteria | | | | |
| *Bordetella pertussis* | 2/11 (18.2) | | 3/579 (0.5) | **66.19 (4.62**, **948.15)** |
| *Chlamydophila pneumoniae* | 0/11 (0.0) | | 16/579 (2.8) | N/E |
| *Haemophilus influenzae* type b | 1/11 (9.1) | | 5/579 (0.9) | **21.68 (1.00**, **468.02)** |
| *Haemophilus influenzae* type b ≥ threshold density ^c^ | 1/11 (9.1) | | 2/579 (0.3) | **9.39 (1.28**, **68.92)** |
| Non-type b *Haemophilus influenzae* | 6/11 (54.5) | | 267/579 (46.1) | 2.90 (0.52, 16.23) |
| Non-type b *Haemophilus influenzae* ≥ threshold density ^c^ | 2/11 (18.2) | | 121/579 (20.9) | 1.13 (0.11, 11.91) |
| *Moraxella catarrhalis* | 5/11 (45.5) | | 384/579 (66.3) | 0.21 (0.03, 1.28) |
| *Mycoplasma pneumoniae* | 0/11 (0.0) | | 3/579 (0.5) | N/E |
| *Streptococcus pneumoniae* | 6/11 (54.5) | | 399/579 (68.9) | 0.51 (0.10, 2.49) |
| *Streptococcus pneumoniae* ≥ threshold density ^d^ | 0/11 (0.0) | | 54/579 (9.3) | N/E |
| Vaccine type *Streptococcus pneumoniae* ^e^ | 0/11 (0.0) | | 21/582 (3.6) | N/E |
| Non-vaccine type *Streptococcus pneumoniae* ^e^ | 0/11 (0.0) | | 35/582 (6.0) | N/E |
| *Streptococcus pneumoniae* in whole blood | 0/8 (0.0) | | 23/225 (10.2) | N/E |
| *Streptococcus pneumoniae* in whole blood ≥ threshold density ^f^ | 1/11 (9.1) | | 31/583 (5.3) | 3.94 (0.32, 47.79) |
| Salmonella spp | 0/11 (0.0) | | 0/579 (0.0) | N/E |
| *Staphylococcus aureus* | 3/11 (27.3) | | 102/579 (17.6) | 1.10 (0.17, 7.13) |
| Fungal species | | | | |
| *Pneumocystis jirovecii* | 3/11 (27.3) | | 65/579 (11.2) | 4.36 (0.75, 25.45) |
| *Pneumocystis jirovecii* ≥ threshold density ^g^ | 2/11 (18.2) | | 19/579 (3.3) | **17.35 (1.92**, **156.83)** |
| Viruses | | | | |
| Any viral pathogen | | 8/11 (72.7) | 440/579 (76.0) | 0.81 (0.21, 3.10) |
| Any viral pathogen, above cut-off density threshold ^b^ | | 7/11 (63.6) | 406/579 (70.1) | 0.70 (0.20, 2.45) |
| Adenovirus | | 2/11 (18.2) | 46/579 (7.9) | **9.39 (1.28**, **68.92)** |
| Human cytomegalovirus | | 3/11 (27.3) | 294/579 (50.8) | 0.34 (0.06, 1.82) |
| Human cytomegalovirus ≥ threshold density ^h^ | | 1/11 (9.1) | 174/579 (30.1) | 0.19 (0.02, 2.16) |
| Coronavirus 229 | | 0/11 (0.0) | 1/579 (0.2) | N/E |
| Coronavirus 43 | | 1/11 (9.1) | 27/579 (4.7) | 3.16 (0.26, 38.00) |
| Coronavirus 63 | | 0/11 (0.0) | 22/579 (3.8) | N/E |
| Coronavirus HKU | | 0/11 (0.0) | 14/579 (2.4) | N/E |
| Influenza A | | 0/11 (0.0) | 10/579 (1.7) | N/E |
| Influenza B | | 0/11 (0.0) | 2/579 (0.3) | N/E |
| Influenza C | | 0/11 (0.0) | 6/579 (1.0) | N/E |
| Human bocavirus | | 1/11 (9.1) | 60/579 (10.4) | 0.07 (0.00, 4.00) |
| Human metapneumovirus A/B | | 1/11 (9.1) | 19/579 (3.3) | 10.37 (0.80, 133.65) |
| Parainfluenza virus 1 | | 1/11 (9.1) | 2/579 (0.3) | **70.89 (1.43**, **3510.98)** |
| Parainfluenza virus 2 | | 0/11 (0.0) | 6/579 (1.0) | N/E |
| Parainfluenza virus 3 | | 0/11 (0.0) | 11/579 (1.9) | N/E |
| Parainfluenza virus 4 | | 0/11 (0.0) | 10/579 (1.7) | N/E |
| Parechovirus/Enterovirus | | 0/11 (0.0) | 42/579 (7.3) | N/E |
| Human rhinovirus | | 3/11 (27.3) | 133/579 (23.0) | 3.02 (0.47, 19.38) |
| Respiratory syncytial virus | | 1/11 (9.1) | 21/579 (3.6) | 2.30 (0.20, 26.95) |

Abbreviations: CI = Confidence Interval; CXR+ = Radiologically-confirmed pneumonia; HIV = Human immunodeficiency virus type-1; N/E = No estimate; NP/OP = Nasopharyngeal/oropharyngeal.

^a^ Conditional odds ratio derived by logistic regression, adjusting age (in months) and presence of all other pathogens: two analyses were combined in the output of this Table: the first with no threshold applied for human cytomegalovirus, *H. influenzae*, *P. jirovecii*, and *S. pneumoniae*, and the second with threshold density cut-offs (as noted below) applied to these pathogens. The first analysis output was used to report the adjusted conditional odds for cytomegalovirus, *H. influenzae*, *P. jirovecii*, and *S. pneumoniae* with no threshold density cut-off applied. The second analysis output was used to report the adjusted conditional odds for all pathogens named in the Table.

^b^ Cut-off density threshold which best distinguished between cases and controls, derived by receiver operating characteristic analysis using leave-one-out cross-validation.

^c^ Cut-off density for *H. influenzae* (non-type b, and type b) on NP/OP swabs: 5.9 log_10_ copies/mL.

^d^ Cut-off density for *S. pneumoniae* on NP/OP swabs: 6.9 log_10_ copies/mL.

^e^ Vaccine-type pneumococcus amongst children with high density NP/OP pneumococcal carriage.

^f^ Cut-off density for *S. pneumoniae* in whole blood specimens: 2.2 log_10_ copies/mL.

^g^ Cut-off density for *P. jirovecii* on NP/OP swabs: 4.0 log_10_ copies/mL.

^h^ Cut-off density for human cytomegalovirus on NP/OP swabs: 4.9 log_10_ copies/mL.
