# Supplementary material for: The Etiology of Pneumonia in HIV-uninfected South African Children: Findings From the Pneumonia Etiology Research for Child Health (PERCH) Study
Source: Pediatr Infect Dis J. 2021 Aug 25;40(9):S59–68. doi: 10.1097/INF.0000000000002650 (PMC8448398; doi:10.1097/INF.0000000000002650)
Supplement: Supplementary file 10 [file inf-40-s59-s010.docx]

***Supplemental Digital Content 10: Conditional Odds Ratios in the Comparison between Cases Dying In-hospital, and Controls: HIV-uninfected Children***

| Pathogen | Cases Dying In-hospital | | Controls | Conditional Odds Ratio (95% CI) ^a^ |
| --- | --- | --- | --- | --- |
|  |  |  |  | Cases Dying In-hospital vs. Controls |
| Any non-viral pathogen | 19/20 (95.0) | | 774/823 (94.0) | 1.25 (0.16, 9.63) |
| Any non-viral pathogen, above cut-off density threshold ^b^ | 17/20 (85.0) | | 677/823 (82.3) | 1.24 (0.36, 4.31) |
| Bacteria | | | | |
| *Bordetella pertussis* | 3/20 (15.0) | | 4/823 (0.5) | **22.01 (3.65**, **132.53)** |
| *Chlamydophila pneumoniae* | 0/20 (0.0) | | 20/823 (2.4) | N/E |
| *Haemophilus influenzae* type b | 1/20 (5.0) | | 7/823 (0.9) | **12.42 (1.05**, **146.66)** |
| *Haemophilus influenzae* type b ≥ threshold density ^c^ | 1/20 (5.0) | | 2/823 (0.2) | 1.90 (0.36, 9.97) |
| Non-type b *Haemophilus influenzae* | 8/20 (40.0) | | 389/823 (47.3) | 1.11 (0.38, 3.29) |
| Non-type b *Haemophilus influenzae* ≥ threshold density ^c^ | 3/20 (15.0) | | 182/823 (22.1) | 0.61 (0.13, 2.94) |
| *Moraxella catarrhalis* | 7/20 (35.0) | | 548/823 (66.6) | *0.20 (0.06*, *0.65)* |
| *Mycoplasma pneumoniae* | 0/20 (0.0) | | 5/823 (0.6) | N/E |
| *Streptococcus pneumoniae* | 11/20 (55.0) | | 568/823 (69.0) | 0.58 (0.20, 1.69) |
| *Streptococcus pneumoniae* ≥ threshold density ^d^ | 1/20 (5.0) | | 80/823 (9.7) | 0.38 (0.03, 4.30) |
| Vaccine type *Streptococcus pneumoniae* ^e^ | 0/20 (0.0) | | 30/826 (3.6) | N/E |
| Non-vaccine type *Streptococcus pneumoniae* ^e^ | 1/20 (5.0) | | 53/827 (6.4) | 0.95 (0.10, 9.02) |
| *Streptococcus pneumoniae* in whole blood | 0/8 (0.0) | | 23/225 (10.2) | N/E |
| *Streptococcus pneumoniae* in whole blood ≥ threshold density ^f^ | 1/20 (5.0) | | 42/827 (5.1) | 1.87 (0.19, 18.55) |
| Salmonella spp | 0/20 (0.0) | | 0/823 (0.0) | N/E |
| *Staphylococcus aureus* | 6/20 (30.0) | | 149/823 (18.1) | 0.94 (0.29, 3.01) |
| Fungal species | | | | |
| *Pneumocystis jirovecii* | 6/20 (30.0) | | 88/823 (10.7) | **4.56 (1.39**, **14.93)** |
| *Pneumocystis jirovecii* ≥ threshold density ^g^ | 4/20 (20.0) | | 25/823 (3.0) | **10.46 (2.42**, **45.17)** |
| Viruses | | | | |
| Any viral pathogen | | 15/20 (75.0) | 613/823 (74.5) | 1.02 (0.36, 2.84) |
| Any viral pathogen, above cut-off density threshold ^b^ | | 14/20 (70.0) | 561/823 (68.2) | 1.05 (0.40, 2.78) |
| Adenovirus | | 2/20 (10.0) | 84/823 (10.2) | 1.90 (0.36, 9.97) |
| Human cytomegalovirus | | 8/20 (40.0) | 378/823 (45.9) | 1.07 (0.39, 2.95) |
| Human cytomegalovirus ≥ threshold density ^h^ | | 5/20 (25.0) | 212/823 (25.8) | 1.16 (0.35, 3.90) |
| Coronavirus 229 | | 0/20 (0.0) | 2/823 (0.2) | N/E |
| Coronavirus 43 | | 1/20 (5.0) | 46/823 (5.6) | 1.08 (0.12, 9.77) |
| Coronavirus 63 | | 1/20 (5.0) | 26/823 (3.2) | 1.93 (0.16, 23.14) |
| Coronavirus HKU | | 0/20 (0.0) | 18/823 (2.2) | N/E |
| Influenza A | | 0/20 (0.0) | 13/823 (1.6) | N/E |
| Influenza B | | 0/20 (0.0) | 3/823 (0.4) | N/E |
| Influenza C | | 0/20 (0.0) | 6/823 (0.7) | N/E |
| Human bocavirus | | 2/20 (10.0) | 83/823 (10.1) | 0.42 (0.06, 3.00) |
| Human metapneumovirus A/B | | 3/20 (15.0) | 27/823 (3.3) | **7.03 (1.56**, **31.63)** |
| Parainfluenza virus 1 | | 1/20 (5.0) | 2/823 (0.2) | **31.31 (1.38**, **712.79)** |
| Parainfluenza virus 2 | | 0/20 (0.0) | 8/823 (1.0) | N/E |
| Parainfluenza virus 3 | | 0/20 (0.0) | 17/823 (2.1) | N/E |
| Parainfluenza virus 4 | | 0/20 (0.0) | 12/823 (1.5) | N/E |
| Parechovirus/Enterovirus | | 1/20 (5.0) | 62/823 (7.5) | 1.72 (0.20, 14.70) |
| Human rhinovirus | | 4/20 (20.0) | 187/823 (22.7) | 0.98 (0.26, 3.66) |
| Respiratory syncytial virus | | 2/20 (10.0) | 27/823 (3.3) | 5.54 (0.98, 31.31) |

Abbreviations: CI = Confidence Interval; CXR+ = Radiologically-confirmed pneumonia; HIV = Human immunodeficiency virus type-1; N/E = No estimate; NP/OP = Nasopharyngeal/oropharyngeal.

^a^ Conditional odds ratio derived by logistic regression, adjusting age (in months) and presence of all other pathogens: two analyses were combined in the output of this Table: the first with no threshold applied for human cytomegalovirus, *H. influenzae*, *P. jirovecii*, and *S. pneumoniae*, and the second with threshold density cut-offs (as noted below) applied to these pathogens. The first analysis output was used to report the adjusted conditional odds for cytomegalovirus, *H. influenzae*, *P. jirovecii*, and *S. pneumoniae* with no threshold density cut-off applied. The second analysis output was used to report the adjusted conditional odds for all pathogens named in the Table.

^b^ Cut-off density threshold which best distinguished between cases and controls, derived by receiver operating characteristic analysis using leave-one-out cross-validation.

^c^ Cut-off density for *H. influenzae* (non-type b, and type b) on NP/OP swabs: 5.9 log_10_ copies/mL.

^d^ Cut-off density for *S. pneumoniae* on NP/OP swabs: 6.9 log_10_ copies/mL.

^e^ Vaccine-type pneumococcus amongst children with high density NP/OP pneumococcal carriage.

^f^ Cut-off density for *S. pneumoniae* in whole blood specimens: 2.2 log_10_ copies/mL.

^g^ Cut-off density for *P. jirovecii* on NP/OP swabs: 4.0 log_10_ copies/mL.

^h^ Cut-off density for human cytomegalovirus on NP/OP swabs: 4.9 log_10_ copies/mL.
