# Supplementary figures and images for: The Etiology of Pneumonia in HIV-uninfected South African Children: Findings From the Pneumonia Etiology Research for Child Health (PERCH) Study
Source: Pediatr Infect Dis J. 2021 Aug 25;40(9):S59–68. doi: 10.1097/INF.0000000000002650 (PMC8448398; doi:10.1097/INF.0000000000002650)

## HEU < 12 months

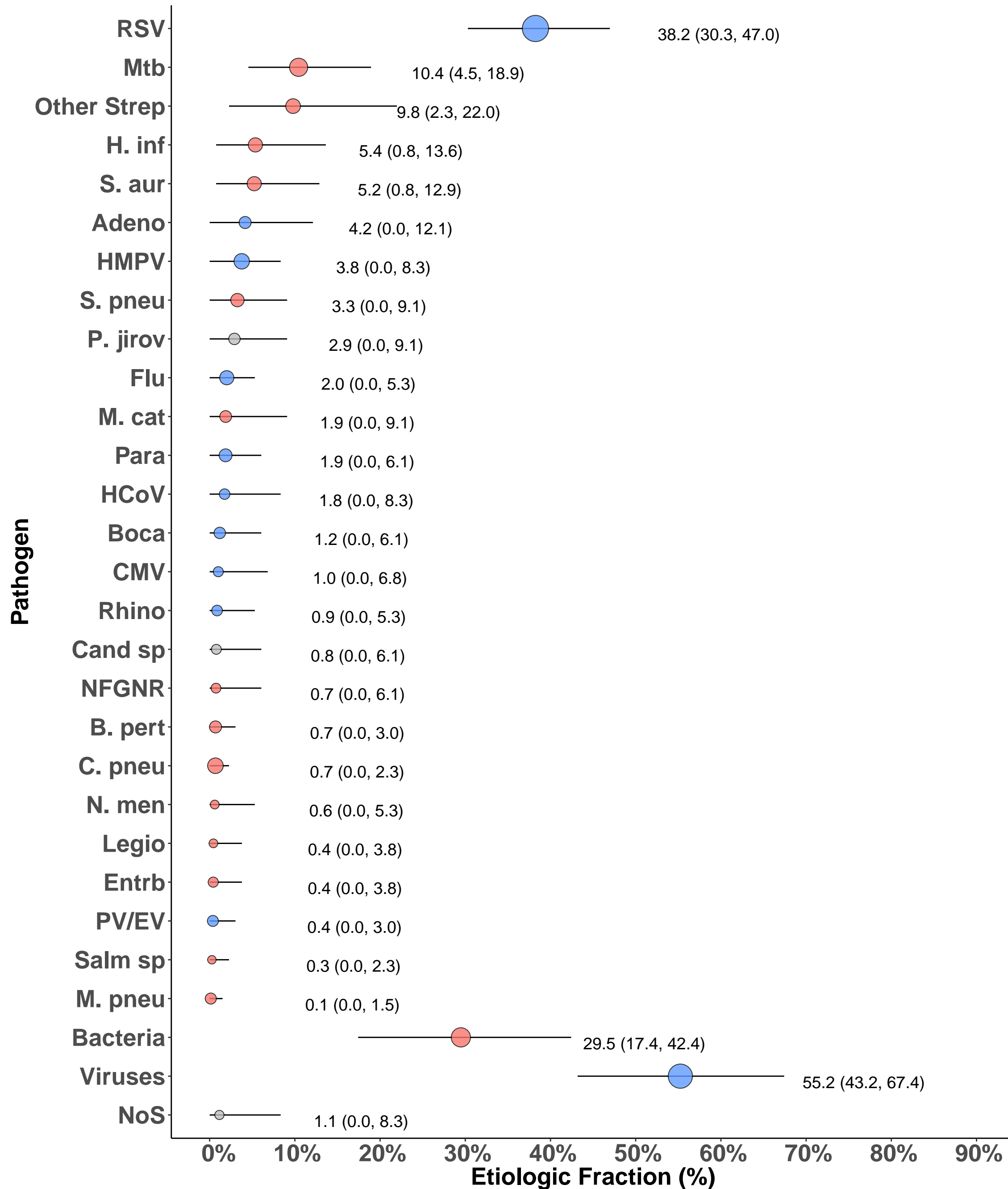

## HEU ≥ 12 months

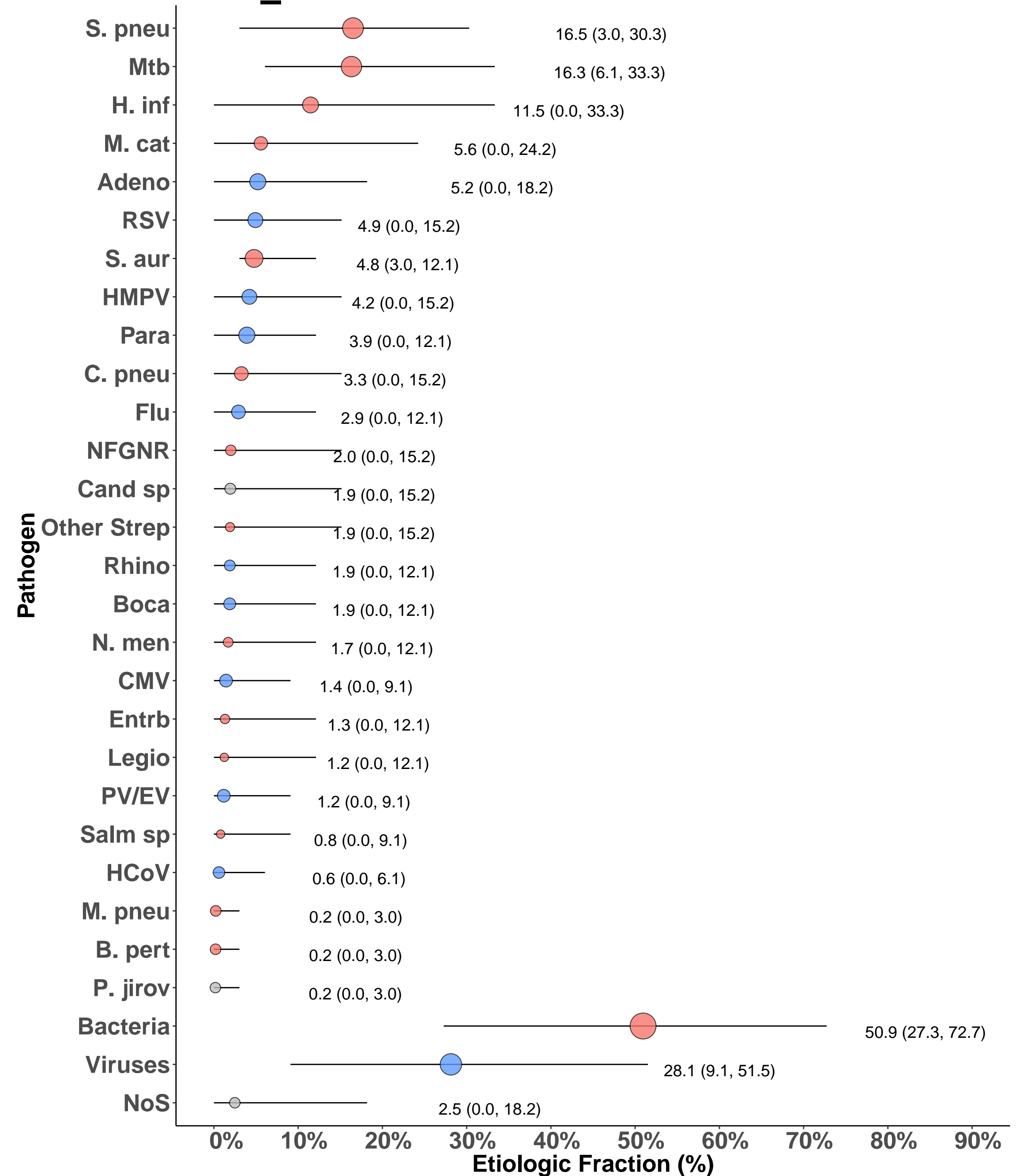

Supplement: Supplementary file 11 [file inf-40-s59-s011.pdf]

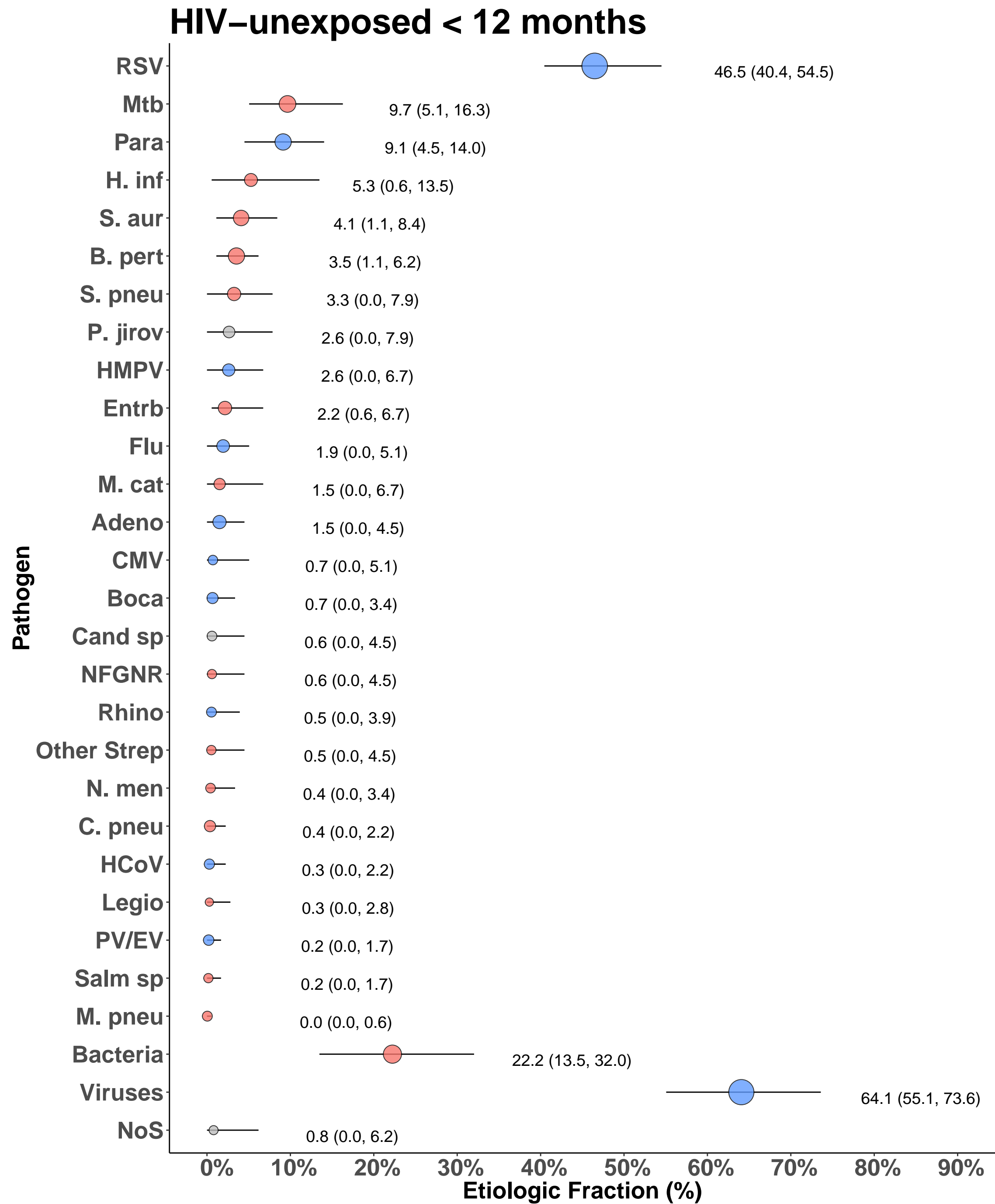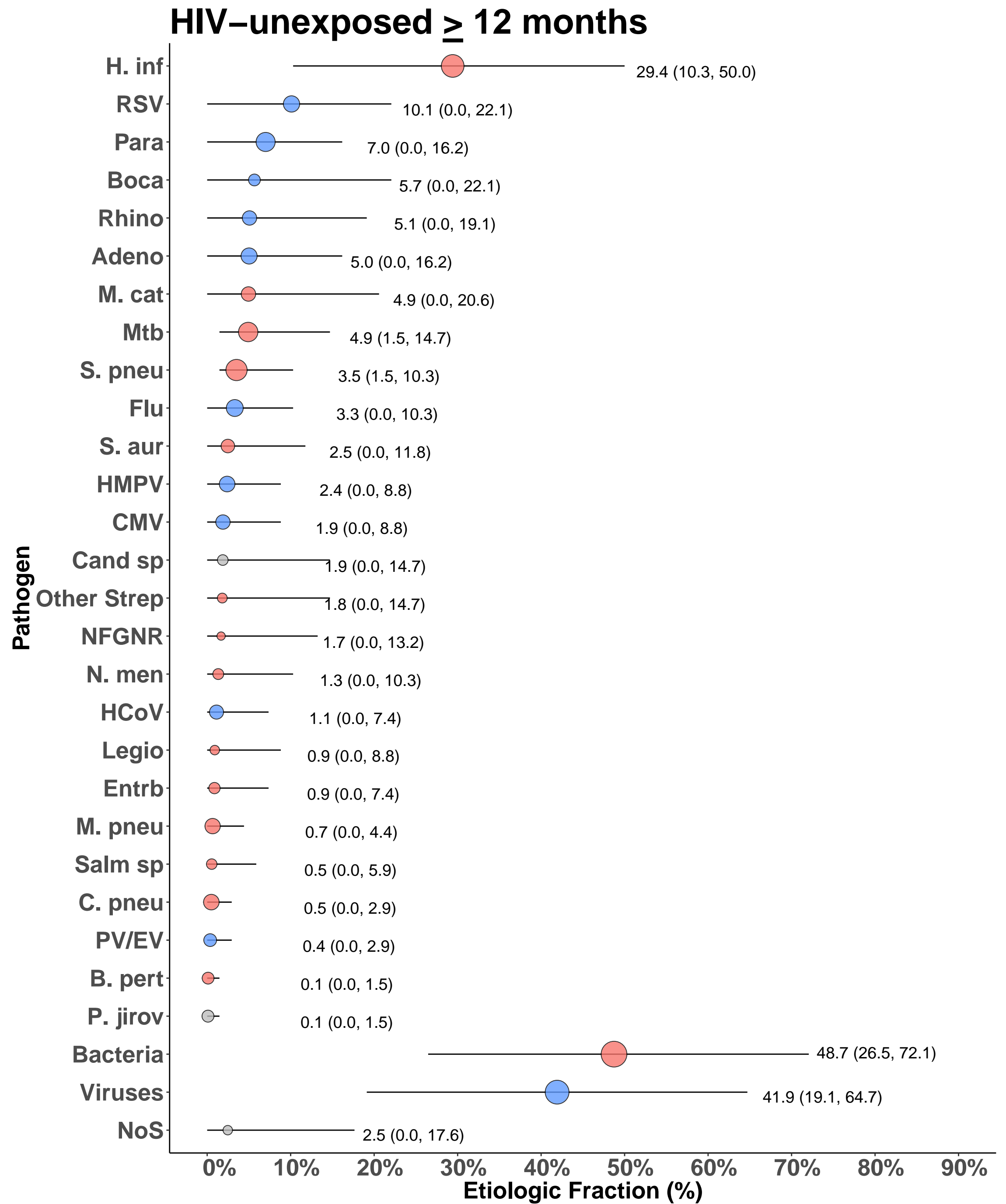

Supplement: Supplementary file 12 [file inf-40-s59-s012.pdf]
