# Supplementary material for: The Etiology of Pneumonia in HIV-uninfected South African Children: Findings From the Pneumonia Etiology Research for Child Health (PERCH) Study
Source: Pediatr Infect Dis J. 2021 Aug 25;40(9):S59–68. doi: 10.1097/INF.0000000000002650 (PMC8448398; doi:10.1097/INF.0000000000002650)
Supplement: Supplementary file 13 [file inf-40-s59-s013.docx]

## Supplemental Digital Content 13: ‘Top 10’ Pathogens Associated with Radiologically-confirmed Pneumonia in HEU and HIV-unexposed Children, Stratified by Pneumonia Severity

|  | HIV-uninfected | | | | HIV-exposed Children | | | | HIV-unexposed Children | | | |
| --- | --- | --- | --- | --- | --- | --- | --- | --- | --- | --- | --- | --- |
|  | Severe | | Very Severe | | Severe | | Very Severe | | Severe | | Very Severe | |
|  | Pathogen | EF (95% CrI) | Pathogen | EF (95% CrI) | Pathogen | EF (95% CrI) | Pathogen | EF (95% CrI) | Pathogen | EF (95% CrI) | Pathogen | EF (95% CrI) |
| **1** | RSV | 36.8 (31.5, 43.1) | RSV | 34.0 (27.8, 41.0) | RSV | 33.1 (25.7, 41.6) | RSV | 31.7 (23.4, 40.6) | RSV | 39.0 (33.1, 46.4) | RSV | 35.9 (28.7, 45.0) |
| **2** | *Mtb* | 10.1 (6.0, 15.7) | *Mtb* | 8.5 (3.5, 15.3) | *Mtb* | 15.5 (7.9, 25.7) | *S. aur* | 11.3 (1.6, 21.9) | Para | 12.1 (7.2, 18.1) | *Mtb* | 11.6 (3.8, 22.5) |
| **3** | Para | 8.1 (4.9, 12.0) | *S. aur* | 7.8 (2.8, 14.6) | Other Strep | 12.6 (3.0, 26.7) | *S. pneu* PCV13 | 10.0 (1.6, 18.8) | *Hi* non-b | 10.5 (3.0, 19.9) | *Hi* non-b | 8.4 (0.0, 23.8) |
| **4** | *Hi* non-b | 8.0 (2.6, 14.6) | *Hi* non-b | 6.9 (0.7, 16.7) | *Hi* non-b | 3.9 (0.0, 13.9) | HMPV | 6.8 (0.0, 14.1) | *Mtb* | 6.8 (3.0, 13.3) | Rhino | 7.2 (0.0, 17.5) |
| **5** | Other Strep | 5.2 (1.1, 10.9) | *S. pneu* PCV13 | 5.1 (1.4, 9.7) | *P. jirov* | 3.5 (0.0, 9.9) | *Hi* non-b | 5.0 (0.0, 17.2) | *S. aur* | 3.6 (0.6, 8.4) | *S. aur* | 4.9 (1.2, 12.5) |
| **6** | Adeno | 2.9 (0.4, 6.4) | Rhino | 4.7 (0.0, 11.1) | *S. pneu* PCV13 | 3.2 (0.0, 7.9) | Para | 4.8 (0.0, 12.5) | Flu | 3.6 (0.6, 7.2) | *S. pneu* Non-PCV13 | 4.8 (0.0, 12.5) |
| **7** | *S. aur* | 2.8 (0.7, 6.0) | Para | 4.7 (0.7, 9.7) | Hib | 3.0 (1.0, 7.9) | *Mtb* | 4.6 (1.6, 12.5) | Adeno | 2.8 (0.0, 7.2) | Para | 4.6 (0.0, 11.2) |
| **8** | Flu | 2.7 (0.7, 4.9) | HMPV | 4.4 (0.7, 9.0) | Adeno | 3.0 (0.0, 9.9) | *M. cat* | 3.5 (0.0, 14.1) | *B. pert* | 2.5 (0.0, 5.4) | HMPV | 2.4 (0.0, 8.8) |
| **9** | *P. jirov* | 2.2 (0.0, 5.6) | *S. pneu* Non-PCV13 | 2.9 (0.0, 7.6) | *M. cat* | 2.2 (0.0, 10.9) | Flu | 3.2 (0.0, 9.4) | HMPV | 2.4 (0.0, 6.6) | *M. cat* | 2.2 (0.0, 11.2) |
| **10** | HMPV | 2.0 (0.0, 5.2) | *M. cat* | 2.8 (0.0, 9.0) | CMV | 1.7 (0.0, 9.9) | Adeno | 2.4 (0.0, 10.9) | Entrb | 2.4 (0.6, 7.2) | Flu | 1.9 (0.0, 7.5) |
|  | **Top 10** | **80.7 (72.3, 88.4)** | **Top 10** | **81.7 (71.5, 91.0)** | **Top 10** | **81.7 (68.3, 92.1)** | **Top 10** | **83.2 (67.2, 95.3)** | **Top 10** | **85.8 (75.9, 94.6)** | **Top 10** | **84.0 (70.0, 95.0)** |

Abbreviations: Adeno = Adenovirus; *B. pert* = *Bordetella pertussis*; CrI = Credible Interval; CMV = Human cytomegalovirus; EF = etiologic fraction; Flu = Influenza virus; HBOV = Human bocavirus; *Hi* non-b = Non-type b *Haemophilus influenzae*; Hib = *Haemophilus influenzae* type b; HIV = Human immunodeficiency virus type-1; HMPV = Human metapneumovirus A/B; Flu B = Influenza B; *M. cat* = *Moraxella catarrhalis*; *Mtb* = *Mycobacterium tuberculosis*; *P. jirov* = *Pneumocystis jirovecii*; Para = Parainfluenza virus; PCV13 = 13-valent pneumococcal conjugate vaccine; Rhino = Human rhinovirus; RSV = Respiratory syncytial virus A/B; *S. aur* = *Staphylococcus aureus*; *S. pneu* Non-PCV13 = Non-13-valent PCV type *Streptococcus pneumoniae*; *S. pneu* PCV13 = 13-valent PCV type *Streptococcus pneumoniae*.

Other Strep includes *Streptococcus pyogenes* and *Enterococcus faecium*.

Radiologically-confirmed defined as consolidation and/or other infiltrate on chest radiograph.
