# Supplementary material for: The Etiology of Pneumonia in HIV-uninfected South African Children: Findings From the Pneumonia Etiology Research for Child Health (PERCH) Study
Source: Pediatr Infect Dis J. 2021 Aug 25;40(9):S59–68. doi: 10.1097/INF.0000000000002650 (PMC8448398; doi:10.1097/INF.0000000000002650)
Supplement: Supplementary file 14 [file inf-40-s59-s014.docx]

## Supplemental Digital Content 14: Sensitivity Analysis Using Lower Sensitivity Priors for M. tuberculosis, and Resulting ‘Top 5’ Pathogens Associated with Radiologically-confirmed Pneumonia in HIV-exposed and HIV-unexposed Children, Stratified by Age and Pneumonia Severity

|  | Sensitivity Prior 10-30% | | | | | | | |
| --- | --- | --- | --- | --- | --- | --- | --- | --- |
|  | HIV-exposed Children | | | | HIV-unexposed Children | | | |
|  | Pathogen | EF (95% CrI) | Pathogen | EF (95% CrI) | Pathogen | EF (95% CrI) | Pathogen | EF (95% CrI) |
|  | Age 1-11 months | | Age 12-59 months | | Age 1-11 months | | Age 12-59 months | |
| **1** | RSV | 37.6 (29.5, 45.5) | *Mtb* | 19.5 (6.1, 36.4) | RSV | 45.7 (39.9, 52.8) | *Hi* non-b | 29.1 (10.3, 50.0) |
| **2** | *Mtb* | 13.9 (6.1, 25.0) | *S. pneu* PCV13 | 11.9 (0.0, 27.3) | *Mtb* | 12.3 (6.2, 19.7) | RSV | 9.3 (0.0, 22.1) |
| **3** | Other Strep | 9.0 (2.3, 20.5) | *Hi* non-b | 7.2 (0.0, 27.3) | Para 3 | 6.8 (2.8, 11.2) | *Mtb* | 7.2 (1.5, 20.6) |
| **4** | *S. aur* | 5.1 (0.8, 12.9) | *M. cat* | 5.2 (0.0, 21.2) | *Hi* non-b | 4.2 (0.0, 11.2) | HBOV | 5.4 (0.0, 20.6) |
| **5** | Adeno | 3.8 (0.0, 11.4) | Adeno | 5.0 (0.0, 18.2) | *S. aur* | 4.0 (1.1, 8.4) | *M. cat* | 4.9 (0.0, 22.1) |
|  | Severe | | Very Severe | | Severe | | Very Severe | |
| **1** | RSV | 32.6 (24.8, 40.6) | RSV | 31.5 (23.4, 40.6) | RSV | 38.7 (32.5, 45.8) | RSV | 35.5 (27.5, 43.8) |
| **2** | *Mtb* | 20.3 (9.9, 32.7) | *S. aur* | 11.4 (1.6, 23.4) | *Hi* non-b | 9.7 (2.4, 19.3) | *Mtb* | 14.3 (5.0, 26.2) |
| **3** | Other Strep | 10.9 (3.0, 23.8) | *S. pneu* PCV13 | 9.7 (1.6, 18.8) | *Mtb* | 9.4 (3.6, 16.9) | *Hi* non-b | 8.2 (0.0, 22.5) |
| **4** | *Hi* non-b | 3.5 (0.0, 12.9) | HMPV | 6.7 (0.0, 14.1) | Para 3 | 6.3 (3.0, 10.8) | Rhino | 7.2 (0.0, 17.5) |
| **5** | *P. jirov* | 3.5 (0.0, 8.9) | *Mtb* | 5.6 (1.6, 15.6) | Para 1 | 5.3 (2.4, 9.0) | *S. aur* | 4.7 (1.2, 11.2) |

Abbreviations: Adeno = Adenovirus; CrI = Credible Interval; EF = Etiologic fraction; HBOV = Human bocavirus; *Hi* non-b = Non-type b *Haemophilus influenzae*; HIV = Human immunodeficiency virus type-1; HMPV = Human metapneumovirus A/B; *Mtb* = *Mycobacterium tuberculosis*; *P. jirov* = *Pneumocystis jirovecii*; Para 1 = Parainfluenza virus 1; Para 3 = Parainfluenza virus 3; PCV13 = 13-valent pneumococcal conjugate vaccine; Rhino = Human rhinovirus; RSV = Respiratory syncytial virus A/B; *S. aur* = *Staphylococcus aureus*; *S. pneu* PCV13 = 13-valent PCV type *Streptococcus pneumoniae*.

Other Strep includes *Streptococcus pyogenes* and *Enterococcus faecium*.

Radiologically-confirmed defined as consolidation and/or other infiltrate on chest radiograph.
